# Towards Metabolomics-Guided Healthy and Anti-Aging Nutrition

**DOI:** 10.3390/metabo16040241

**Published:** 2026-04-01

**Authors:** Petr G. Lokhov, Elena E. Balashova, Dmitry L. Maslov, Oxana P. Trifonova, Arthur P. Lokhov, Alexander I. Archakov

**Affiliations:** 1 Institute of Biomedical Chemistry, Pogodinskaya St. 10, Moscow 119121, Russia; 2 Department of Mathematical Support and Standardization of Information Technologies, MIREA—Russian Technological University, Moscow 119454, Russia

**Keywords:** aging, metabolomic fingerprinting, diseases, precision nutrition, healthspan, diet

## Abstract

**Background**: Metabolomic studies have generated extensive data on metabolic changes in aging and disease, yet translating this data into practical nutrition guidelines remains challenging. Recent analysis identified pathways common to both processes, termed the metapathway. As a network, it features key central metabolites that most representatively reflect its state. The manageable number of these key metabolites provides a practical basis for translating complex metabolomic data into actionable nutritional information. **Methods:** We developed a conceptual framework for precision nutrition approach involving: (1) selecting an initial (baseline) diet with minimal impact on key metapathway metabolites, (2) defining dietary modifications using foods and supplements that are capable of elevating them, and (3) implementing mass spectrometry-based metabolome fingerprinting to assess individual responses. This capability was evaluated using blood plasma and dried blood spot samples. **Results:** A promising precision nutrition was created, consisting of a selected baseline diet and its metabolomics-guided modification. The metabolic fingerprinting demonstrated the possibility of determining the diet outcome by identifying biological age change with an accuracy of 1 month. **Conclusions**: The fully metabolomics-guided nutrition strategy has been developed and is ready for further human testing to validate its translational potential and health benefits.

## 1. Introduction

An important aspect of medicine is maintaining human health, thereby increasing lifespan. Worldwide, this has led to a continuous rise in life expectancy [1], a trend associated with population aging and a growing burden of aging-related diseases [2,3]. To effectively extend lifespan and mitigate the negative effects of aging, it is necessary to apply discoveries obtained at the molecular level [4,5].

Aging involves changes across all molecular levels, from the genome to the metabolome. It is known that longevity is heritable, with heritability ranging from 20% to about 50% [6,7,8,9,10]. However, lifespan is also influenced by numerous “post-genomic” factors [11,12,13,14]. These include epigenetic alterations, loss of protein (proteostasis) and stem cell (stem cell exhaustion) function, altered intercellular communication (inflammation), cell senescence, and a deregulated response to nutrients (nutrient sensing) [15]. For instance, epigenetic changes, specifically DNA methylation patterns, are closely related to person’s age, enabling the development of accurate epigenetic clocks [16]. Yet, these epigenetic data have limited power in predicting individual health outcomes and time of death. The next, phenotype level, exhibits this property more strongly [16]. Phenotypic clocks, which use readily measurable biological markers to quantify aging and disease-related mortality, can predict mortality more accurately than chronological age itself. 

The molecular phenotype is primarily represented by metabolites: low-molecular-weight compounds that serve as substrates, intermediates, and products of metabolic processes. Collectively, these metabolites constitute the metabolome [4,17]. Metabolomic analysis can identify markers of aging and describe their relationship with pathological processes [18,19,20,21,22]. Databases such as the Human Metabolome Database (HMDB) [23] and MetaboAge [24] compile key findings from these studies, cataloging age-related metabolites and their associated pathways. By detecting variations in metabolites, metabolomics guides interventions and helps assess biological age [22]. Thus, for developing health and anti-aging interventions, including healthy nutrition, and for assessing the body’s response, the metabolomic level appears to be the most promising among all levels of molecular organization.

Alongside physical activity, stress management, and avoiding harmful habits, healthy nutrition is a cornerstone of longevity [25,26,27]. In recent years, the concept of precision nutrition has evolved, which involves tailoring specific dietary recommendations based on an individual’s metabolism [28]. The potential of using metabolomics to inform such personalized nutrition strategies has already been demonstrated [29,30,31,32,33], including applications in food intake biomarker discovery, monitoring metabolic responses to dietary interventions, assessing the health impacts of specific foods, and identifying subgroups (metabotypes) for personalized dietary advice based on metabolomic profiles [32,34,35]. Therefore, leveraging modern metabolomic advances to assess health and slow down aging through precision nutrition is highly relevant.

However, the direct application of metabolomic data in human nutrition by scientists, physicians, and nutritionists is not straightforward. Translational research, which serves as a bridge between fundamental science and practical medicine [36,37,38], is required to transform the abundance of available data into an applicable nutritional guidance. Therefore, selecting metabolomics findings with translational potential is the first step.

**Figure 1:**
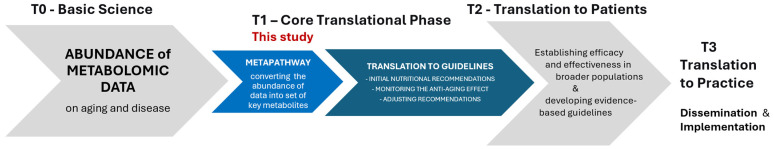
Translational workflow for converting metabolomic data on aging and disease into practical nutrition recommendations. T—phases of translational research. This study is in T1-phase.

Recently, a comparative analysis of untargeted metabolomics studies of aging across various animal models (from nematodes to mammals) and humans has been conducted [39]. The results showed that metabolites significantly differing between age groups are related to carbohydrates, amino acids, carnitines, biogenic amines, and lipids. Notably, the age-associated metabolites identified across different models are largely linked to the same metabolic pathways. This commonality at the metabolomic level has prompted further study of the metabolic connection between aging and disease. It has been established that human aging and pathological conditions affect identical metabolic pathways with a high probability (*p*-value = 0.9996) [40]. By combining these pathways, a single pathway, termed metapathway, was compiled, which captured changes simultaneously linked to health and aging. The identification of this metapathway holds translational potential for developing health and anti-aging interventions, including nutritional strategies [41]. Figure 1 illustrates our translational approach, which uses the identified metapathway to transform accumulated metabolomic data on aging and disease into practical nutrition recommendations.

This study aligns with the T1-phase of translational research [42]. Using the “metapathway” concept, which consolidates age- and disease-related metabolic changes, we convert the abundance of metabolomic data into a limited number of key metabolites. Measuring and interpreting this focused subset is significantly simpler and more practical than analyzing the full, original dataset. This simplification enables the development of practical, metabolomics-based nutritional guidelines.

Thus, the aim of this work is to design a conceptual framework for healthy and anti-aging metabolomics-guided precision nutrition. This framework integrates initial, metabolomics data-based dietary prescriptions with ongoing monitoring of metabolic response and guided dietary adjustments using metabolomic fingerprinting.

## 2. Methods

### 2.1. Scientific Background for Translational Study

In a prior study [40], a meta-analysis of six human untargeted metabolomic studies on aging [43,44,45,46,47,48] and metabolomic data for pathological conditions from the Human Metabolome Database (HMDB) was performed. Combinatorial analysis revealed a significant overlap between metabolic pathways associated with human aging and those linked to pathological conditions. This association was highly non-random, with a high probability (*p* = 0.9996) that the seven identified aging-related pathways were presented among disease-related pathways.

These findings demonstrate a high degree of identity between aging and disease at the metabolic level and allow to identify a set of seven metabolic pathways that simultaneously reflect aging-related and health-related changes:

1. Arginine biosynthesis pathway;
2. Valine, leucine, and isoleucine biosynthesis pathway;
3. Alanine, aspartate, and glutamate metabolism pathway;
4. Butanoate metabolism pathway;
5. Glyoxylate and dicarboxylate metabolism pathway;
6. Phenylalanine, tyrosine, and tryptophan biosynthesis pathway;
7. Aminoacyl-tRNA biosynthesis pathway.

**Figure 2:**
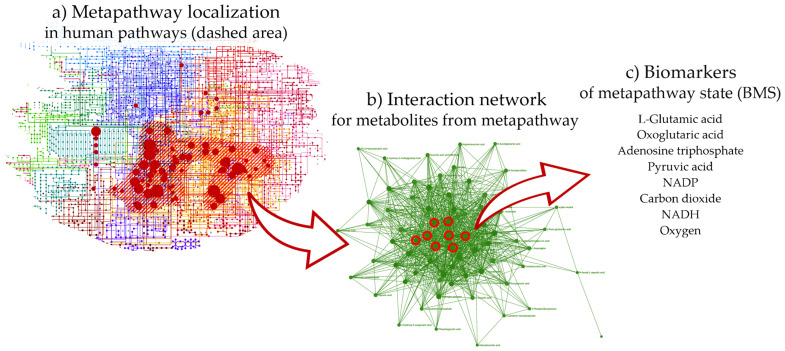
Health and aging-related metapathway and its state biomarkers. (**a**) Location of metapathway’s metabolites within the KEGG global pathway network. Metabolites are highlighted in red; circle size represents the number of associated metabolites. The dashed area indicates the metapathway’s region within human metabolism. Generated using MetaboAnalyst. (**b**) Metabolite–metabolite interaction network for the metapathway. Node size corresponds to the degree of the node (metabolite). Central node metabolites (O), identified as having the highest degree (see Table 1), were selected as biomarkers of metapathway state (BMS). (**c**) The resulting list of BMS. Adapted from [40].

Therefore, in prior work, these pathways were consolidated into a single, integrated metapathway (Figure 2a) providing the basis for the practical application of the original metabolomic data. As a network, the metapathway contains key nodal metabolites that are highly representative of its overall state. These metabolites were termed biomarkers of metapathway state (BMS). Manipulating this limited set of metabolites is feasible for practical application. Thus, in the present work, the metapathway existence was considered a scientific background, and nutritional guidance was designed around the known or expected effects of nutrients on its central node metabolites.

Precision nutrition uses a dynamic approach, where dietary recommendations are adjusted based on an individual’s response, rather than a static plan applied to a wide group. To enable this, metabolic fingerprinting was proposed to monitor changes in biological age following dietary modification. The selection of specific foods to modulate BMS levels in the body is suggested based on the fundamental principle of nutritional science: consuming foods enriched with the target compounds or their precursors to increase BMS levels, while vice versa to decrease them.

### 2.2. Selection of Biomarkers of Metapathway State (BMS)

To select BMS, a metabolite–metabolite interaction network previously constructed for the metapathway was used (Figure 2b) [40]. To build the metabolite–metabolite interaction network and determine the degree of its nodes, the network analysis module of the MetaboAnalyst 6 (option ‘metabolite–metabolite interaction network’; layout Fruchterman–Reingold; accessed on 8 September 2024) was used. The chemical–chemical associations for the metabolites were extracted from STITCH [49] so that only confident interactions were used for this. The central nodes of network were considered key metabolites (Table 1) that most effectively reflect the state of the overall metapathway [50]. Essentially, metabolites that participate in the greatest number of reactions occurring in the metapathway were selected, which makes them the best representatives of the metapathway state. To define central nodes, the degree, as the direct metric to characterize the centrality of nodes in network analysis, was used. Metabolites with the highest centrality, i.e., central nodes, were identified as BMS, which can be used to monitor the metapathway state and to select foods, supplements, and a baseline diet for precision nutrition design.

### 2.3. Scientific Evidence Linking BMS to Health and Longevity

To confirm that metabolomics-derived BMS indeed reflect health and longevity, the published literature was reviewed for supporting evidence.

### 2.4. Implementation of Metabolomics-Guided Precision Nutrition

Precision nutrition is an individualized approach that involves assessing a person’s response to a diet and then adjusting it accordingly. In this framework, the initial baseline diet is modified using selected foods and supplements. To quantify the individual’s response, the use of blood plasma metabolome fingerprinting to measure changes in biological age was proposed.

### 2.5. Selection of a Baseline Diet

A baseline diet for metabolomics-guided nutrition was selected from an analysis of popular diets, including the Mediterranean, ketogenic, paleolithic, and vegan diets, as well as intermittent fasting. The selection prioritized evidence from human studies over animal or theoretical data. The chosen diet was required to have the smallest effect on BMS. Furthermore, any observed effect on BMS had to be beneficial and weaker than the effects of other diets.

### 2.6. Selection of Foods and Food Supplements for Modifying the Baseline Diet

To modify the baseline diet, it is proposed to incorporate specific foods and food supplements rich in BMS or their metabolic precursors, which is in line with the standard nutritional approach to diet formulation [51], specifically, increasing the level of target compounds in the body through the consumption of foods enriched with such compounds or their precursors. Since the choice of the baseline diet is based on minimizing the impact on BMS levels, adding foods and supplements to the baseline diet is aimed specifically at increasing them in organism. Dietary changes aimed at reducing BMS levels were not considered because they are more difficult to implement and are not consistent with scientific data indicating the advisability of increasing their levels in the organism (see Section 3.5).

### 2.7. Assessment of Nutrition Outcome by Metabolome Fingerprinting

Blood metabolome fingerprinting using direct mass spectrometry was selected as the method for routine monitoring of nutrition outcomes. In this case, the fingerprint is formed by a set of mass spectrometric peak intensities and is characteristic for the individual. The fingerprint’s characteristics, specifically its changes with age, were established in a cohort of patients of varying ages and genders. Furthermore, the feasibility of using this fingerprinting approach for assessing biological age, specifically its precision, technical variability, and biological reproducibility, were evaluated.

#### 2.7.1. Blood Samples

Blood plasma samples (Set 1) from Caucasian healthy subjects (*n* = 190) of different ages (from 18 to 81 years old) and body mass index (BMI) in the normal range were taken from the previously conducted metabolomic study [52] (Table 2).

Blood plasma samples from Caucasian healthy subjects used to construct an age-related metabolic curve for the “metabolic clock” testing (Set 2: curve-building samples including 30 samples obtained from the same subjects at intervals of 3, 6, 12, and 18 months) were taken from the previous study [53]. Dried blood spot samples (Set 3, Caucasian subjects) were taken from another previously conducted study [54,55]. Dried blood spot samples (Sets 4, 5, and 6) were provided by Caucasian healthy volunteers of normal BMI after signing informed consent and permission to publish personal metabolomic data. For sample Set 6, a man and a woman provided blood samples on an empty stomach and 1 h after the morning meal (mashed potatoes (200 g, composition: potatoes, milk (2.5%), butter, salt), cutlet (100 g, composition: ground meat (pork/beef), onion, wheat bread, chicken egg, salt, pepper), and tea with sugar (200 mL)).

The characteristics of the subjects for sample sets are presented in Supplementary Materials (Tables S1–S4).

#### 2.7.2. Blood Plasma Sample Preparation

Blood samples were collected in the morning after overnight fasting into EDTA Vacutainer plasma tubes (BD, Franklin Lakes, NJ, USA) and cooled down at 4 °C immediately. Blood plasma was separated by centrifugation according to the manufacturer’s instructions (4000 rpm for 10 min at 4 °C), transferred into a clean 2 mL Eppendorf, and immediately stored at −80 °C until analysis. For analysis, the frozen plasma samples were thawed on ice, and an aliquot (10 µL) was mixed with 80 µL pre-cooled methanol (J.T. Baker, Gliwice, Poland) and 10 µL water (Sigma-Aldrich, St. Louis, MO, USA). The mixture was incubated for 10 min (on ice with periodical shaking) and centrifuged (13,000× *g*, 4 °C, 15 min). The supernatant was transferred to a clean 2 mL Eppendorf, and 10 µL of the supernatant was mixed with fifty volumes of methanol containing 0.1% formic acid (Fluka, Munich, Germany). As an internal standard, 0.4 µL (5 mg/L) of losartan solution was added. The resulting solutions were analyzed by direct infusion mass spectrometry.

#### 2.7.3. DBS Samples Preparation

For metabolite extraction, the circles with dried blood spots (Whatman™ 903 Proteinsaver Snap-Apart Card, Cytiva, Marlborough, MA, USA) were cut out and divided in half, one part of which was placed in clean Eppendorf ™ tubes, where 40 µL of water (LiChrosolv; Merck KGaA, Darmstadt, Germany) and 160 µL of methanol (Fluka, Munich, Germany) were added and mixed. After 10 min of incubation at room temperature, samples were centrifuged at 13,000× *g* (Centrifuge 5804R; Eppendorf AG, Hamburg, Germany) for 15 min. The supernatant was then transferred to clean plastic Eppendorf ™ tubes, and fifty volumes of methanol containing 0.1% formic acid (Fluka) were added to each tube. The resulting solutions were subjected to direct mass spectrometry analysis.

#### 2.7.4. Mass Spectrometry

Samples were analyzed by direct infusion mass spectrometry (DIMS) using a hybrid quadrupole time-of-flight mass spectrometer (maXis Impact, Bruker Daltonics, Billerica, MA, USA) equipped with an electrospray ionization (ESI) source. The mass spectrometer was set up to prioritize the detection of ions with a mass-to-charge ratio (*m*/*z*) ranging from 45 to 900, with a mass accuracy of 1–3 parts per million (ppm). The spectra were recorded in the positive ion charge detection mode. The samples were injected into the ESI source using a glass syringe (Hamilton Bonaduz AG, Bonaduz, Switzerland) connected to a syringe injection pump (KD Scientific, Holliston, MA, USA). The rate of sample flow to the ionization source was 180 µL/h. Mass spectra were obtained using DataAnalysis version 4.1 (Bruker Daltonics) to summarize one-minute signals.

#### 2.7.5. Mass Spectra Processing

Peak detection, recalibration, and peak intensity calculation of mass spectra were carried out automatically by DataAnalysis software. Masses of compounds were determined from the mass spectrum peaks obtained using the following parameters: peak width, 2; signal-to-noise ratio, 1; relative and absolute threshold intensity, 0.01% and 100, respectively. For recalibration of all the peak *m*/*z* values, the internal standard losartan (*m*/*z* 423.169) was used. Alignment of the *m*/*z* values of the mass peaks between different mass spectra was performed as described previously [56]. The alignment algorithm used was previously specially developed and tested for the high-resolution mass spectra of blood metabolites obtained by DIMS and implemented as an iterative process based on the detection of correlation of mass spectrometry peak patterns.

### 2.8. Age-Related Trajectory of the Blood Metabolome

To obtain the age-related trajectory of the blood plasma metabolome, a $\bar{Z}$-score curve was constructed. To do this, age-related mass peaks were first identified (feature extraction). For the mass peak intensities having non-zero values in at least 15% of the mass spectra, the Spearman correlation probability was calculated (*p*-values for testing the hypothesis of no correlation against the alternative hypothesis of a nonzero correlation; *corr* function, Matlab ver. R2019b, MathWorks, Natick, MA, USA). Mass spectrometry peaks positively and negatively correlated with age (adjusted *p*-value < 0.01; FDR adjusted by *mafdr* function) were converted to Z-scores (3245 peaks for men and 1838 peaks for women). The Z-score is a common way of representing data on a unitless scale and is the data minus the mean divided by the standard deviation of the data. The data with Z-scores were sorted by the age of the subjects, and the Z-scores in the direction of age were smoothed by averaging over 7 years (moving average with a sliding window of ±3 years). The smoothed Z-scores for each subject were averaged, and the resulting values ($\bar{Z}$-scores) were plotted according to the subject’s age. Since many subjects were of the same age, the average of their $\bar{Z}$-scores was plotted. To build a curve for plotted data, a smoothing spline with piecewise polynomial was used (Curve fitting toolbox ver. 3.5.10, Matlab). To demonstrate the stability of the $\bar{Z}$-score curve shape depending on its construction parameters, the adjusted *p*-value was varied, which affects feature extraction efficacy and the number of mass spectrometric peaks used in calculating the $\bar{Z}$-score curve (Figure S1). To exclude the influence of missing values on the $\bar{Z}$-score curve shape, the percentage of mass spectrometry peaks with non-zero intensity values used to construct the curve was calculated (Figure S2).

Mass spectra for DBS samples from sample Set 2 were used to construct an age-related trajectory of the whole blood metabolome in a similar manner.

### 2.9. Biological Age Change Determination

The Euclidean distances from an individual’s metabolic fingerprints to points on the age-related metabolomic curve were measured (*pdist* function, Matlab). Where the similarity is greatest, the resulting distance curve has a minimal value and indicates the biological age of the individual. Given that the age-related curve has a resolution of 1 year, polynomial extrapolation (empirically selected) from three points forming the minimum of the distance curve was used to accurately determine biological age. To determine the change in biological age, the difference between two points of time was measured. The quality of biological age change measurement by the proposed way was validated by chronological age (“metabolic clock” test). For this, an age-related curve was built (sample Set 2, *n* = 124), and changes in biological age for samples collected at intervals of 3, 6, 12, and 18 months (sample Set 2.1; *n* = 30) were compared with changes in chronological age. When using samples from Set 2.1, they were excluded one by one from Set 2 to eliminate their influence on the age-related curve construction. The coefficient of determination of linear extrapolation was used as a criterion for the coincidence of changes in biological and chronological ages.

Technical variability for biological age measurement was assessed by acquiring mass spectra ten times from the same blood plasma sample (male, 36 years old; sample Set 1.1), and the standard deviation, standard error of the measurement (SEM), and mean absolute difference (MAD) were defined.

To describe the use of DBS for measuring biological age change, an age-related metabolomic curve was constructed using DBS samples (Set 3; *n* = 100), and DBS samples were used to measure changes in biological age (Set 4, *n* = 6; samples from women aged 25 and 29 years collected with a gap of three months and one week). Samples collected with a gap of one week from the same women (sample Set 5; *n* = 6) were used to measure biological reproducibility of the DBS approach for measuring biological age. The sample Set 7 was used to assess food intake influence on biological age measurement.

The minimum detectable change (MDC) of biological age change at a 95% confidence level represents the smallest change in a measurement that is unlikely to have occurred due to random error (i.e., precision of measurement). MDC was calculated as 1.96 × $\sqrt{2}$ × SEM, where 1.96 is a statistical constant for the 95% confidence level [57].

## 3. Results

### 3.1. Scientific Evidence Linking BMS to Health and Longevity

**Figure 3:**
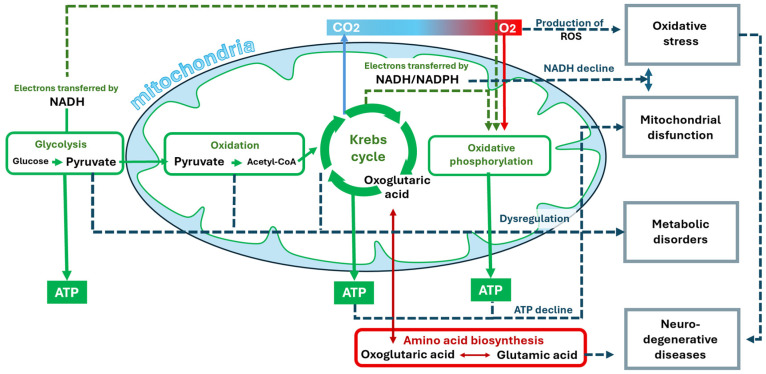
Participation of biomarkers of metapathway state (BMS: glutamic acid, oxoglutaric acid, ATP, pyruvate, NADPH, NADH, CO_2_, and O_2_) in metabolic processes and their relation to disease. The figure was drawn based on the review of published data. The figure shows the deep integration of BMS into central metabolic processes and confirms their connection with the development of diseases.

**Figure 4:**
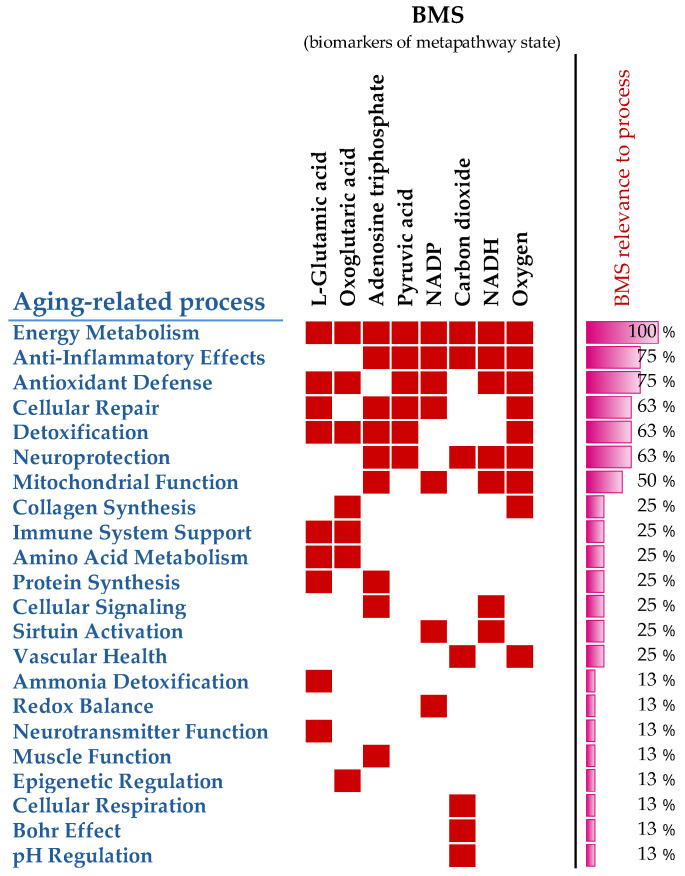
Participation of biomarkers of metapathway state (BMS) in various aging-related processes. The data presented in the figure show the high involvement of BMS in all main processes associated with aging. The figure is based on an analysis of published data collected in Appendix A.

To confirm that metabolomics-derived BMS are associated with health and longevity, the published literature was reviewed, and the supporting evidence was summarized. Figure 3 shows the involvement of BMS in metabolic processes and their relation to disease. Scientific evidence for the involvement of BMS in aging-related processes is summarized in Figure 4, which confirms that metabolomics-derived BMS are integrated in the main aging-related events in the organism. The data underlying this figure are presented in the Appendix A.

### 3.2. Selection of a Baseline Diet from Popular Diets

To implement precision nutrition, a baseline diet must be selected for subsequent adjustment based on metabolic response. For this purpose, we focused on widely recognized diets—the Mediterranean, ketogenic, paleolithic, and vegan diets, as well as intermittent fasting—as their extensive literature allows for a systematic assessment of their effects on BMS. The following several sections provide a brief description of these diets, with a focus on their documented effects on BMS.

#### 3.2.1. Mediterranean Diet

The Mediterranean diet emphasizes the consumption of whole foods, such as fruits, vegetables, whole grains, legumes, nuts, olive oil, and moderate amounts of fish, poultry, and dairy [58]. Red meat and processed foods are limited. The Mediterranean diet might mitigate the imbalance of glutamine and glutamate and associated risk of disease. A case-cohort study (*n* = 892) found that one year of the Mediterranean diet did not significantly alter glutamate levels compared to a control diet [59]. A separate metabolomic study identified pyruvic acid as one of the top five most variable biomarkers in response to the same diet, showing a −21% change over one year [60].

While direct experimental measurements of other BMS changes under the Mediterranean diet are lacking, indirect evidence suggests its components can modulate these key metabolites. For instance, polyphenols and unsaturated fats may support NAD biosynthesis by providing precursors (e.g., tryptophan, vitamin B3) or activating sirtuins [61,62]. The diet may also enhance mitochondrial efficiency and reduce oxidative stress, thereby indirectly promoting ATP production [61,63]. Specific components like extra virgin olive oil, nuts, and berries have been shown to improve mitochondrial respiration and reduce reactive oxygen species (ROS) [61,64].

Although a direct effect of the Mediterranean diet on NADPH levels has not been established, its documented ability to reduce oxidative stress and inflammation may indirectly support the function of NADPH-dependent physiological systems [65].

From an environmental perspective, numerous studies have quantified that higher adherence to the Mediterranean diet is associated with lower dietary CO_2_ emissions, typically in the range of 0.9–6.88 kg CO_2_/day [66,67]. It is important to note that this refers to environmental carbon footprint, not internal physiological CO_2_ levels (e.g., blood partial pressure of CO_2_).

Regarding O_2_, several studies report that the Mediterranean diet increases maximal oxygen consumption (VO_2_max) [68,69]. This improvement in VO_2_max serves as an indirect indicator of enhanced oxygen utilization. Therefore, the diet appears to promote efficient cellular respiration.

#### 3.2.2. Ketogenic Diet

The ketogenic diet is high in fat and very low in carbohydrates (usually less than 50 g per day) to induce ketosis, in which the body burns fat for fuel instead of carbohydrates [70]. Rapid weight loss is common with this diet. Human data on glutamate levels under the ketogenic diet are limited. Existing evidence, derived solely from cerebrospinal fluid studies in epilepsy patients, shows no significant change in glutamate [71]. However, this may not reflect the general population. In fact, research indicates that ketone body metabolism can supply up to 30% of the brain’s glutamate, potentially increasing its release from the nervous system [72]. This is consistent with animal studies, where findings are mixed: some report no change in extracellular glutamate [73], while others show an increase in overall brain tissue glutamate [74,75]. Direct measurements of BMS like oxoglutarate and pyruvate in humans are still an emerging and data-scarce area. It is hypothesized that oxoglutaric acid levels may fluctuate as the Krebs cycle adapts to ketosis and decreased glucose availability [76]. 

As glycolysis decreases due to low carbohydrate intake, pyruvate levels decline. ATP production may drop initially during the adaptation phase but can later increase with enhanced fat oxidation [77]. Consistent with this, the ketogenic diet has shown a significant increase in brain ATP levels in rodent models [78].

Most data on NADH during the ketogenic diet are derived from brain tissue (hippocampus or occipital lobe), while systemic measurements in blood are lacking. Studies in both rodent models and humans consistently show that the diet increases the NAD/NADH ratio in the brain [79]. This shift is supported by a recent ^31^P-MRS study in healthy humans, where a ketogenic drink significantly increased brain NAD levels by 3.4% and decreased NADH by 13%, resulting in an 18% increase in the NAD/NADH ratio [80]. Furthermore, a ketone body, such as β-hydroxybutyrate, can induce antioxidant defense by reducing the cytoplasmic NADP/NADPH ratio [81,82,83]. Collectively, these changes in NAD redox states are linked to neuroprotective and metabolic benefits.

The ketogenic diet reduces CO_2_ levels in the body, primarily through metabolic acidosis and increased respiratory excretion. These effects have been observed in both animal models and humans [84,85]. A 20-day ketogenic diet affected respiratory parameters in healthy individuals by reducing carbon dioxide production (VCO_2_) and end-tidal CO_2_ concentration (PETCO_2_) with *p* < 0.05 [85].

#### 3.2.3. Paleolithic Diet

The paleolithic diet mimics the eating patterns of ancient humans by focusing on whole foods, such as lean meats, fish, fruits, vegetables, nuts, and seeds while excluding modern processed foods, grains, dairy, and legumes [86]. To date, no direct experimental data exist on the diet’s effects on BMS. This gap in the literature is likely because existing research has prioritized broader health outcomes over detailed metabolic profiling. 

Based on its nutritional composition, several indirect effects on BMS can be hypothesized. The diet’s high protein content (from meat and fish) is expected to maintain adequate levels of glutamic acid [87]. Impact on oxoglutaric acid levels is likely to be indirect, stemming from changes in the availability of substrates for the Krebs cycle. 

Furthermore, moderate carbohydrate intake from fruits and vegetables results in improving insulin sensitivity and stabilizing blood sugar levels, thereby promoting balanced pyruvate levels. Enhanced ATP production is plausible through the metabolism of proteins and fats [88], while Krebs cycle function and NADH production are likely sustained. The diet may indirectly influence NADP levels through its impact on oxidative stress [89,90]. Collectively, these factors are posited to support efficient cellular respiration. In its dietary structure, the paleolithic diet is less restrictive than the ketogenic diet, but more restrictive than the Mediterranean diet, with a characteristically higher in protein and lower in carbohydrate profile.

#### 3.2.4. Vegan Diet

The vegan diet excludes all animal products, including meat, dairy, eggs, and honey, and is composed exclusively of plant-based foods, including fruits, vegetables, grains, nuts, seeds, and legumes [91]. The diet is high in complex carbohydrates (fruits, vegetables, grains, and legumes provide stable glucose for energy). Excess fiber slows down digestion and promotes stable blood sugar levels. The diet can improve insulin sensitivity and reduce inflammation, supporting a healthy metabolism [92]. Unlike the ketogenic diet, it relies heavily on carbohydrates for energy. Adequate levels of glutamic acid come from plant proteins (legumes, nuts, seeds) [93]. A study comparing vegans and omnivores found that vegans had higher plasma glutamate levels (+13.1%) compared to omnivores [94]. Another study observed a progressive decrease in plasma glutamic acid levels combined with an increase in glutamine levels with a partially plant-based diet (including fish) [95].

Direct evidence regarding the diet’s impact on other BMS is limited. However, its metabolic profile allows for several inferences. As the diet relies heavily on carbohydrates for energy, pyruvate levels are likely to be stable. Furthermore, the abundant supply of substrates from fruits, vegetables, and grains is expected to support the Krebs cycle, promoting balanced levels of oxoglutarate. Consequently, NADH production from carbohydrate metabolism is likely enhanced, supporting efficient ATP production [96]. The diet’s high content of antioxidant-rich foods may help maintain high NADPH levels, crucial for redox defense [89,90]. The combined effects of high fiber, antioxidants, and efficient substrate utilization are also posited to contribute to efficient cellular respiration.

Despite the lack of data on the dynamics of physiological CO_2_ (e.g., blood pCO_2_, bicarbonate, or end-tidal CO_2_) in response to the vegan diet, many studies have quantified the carbon footprint of the vegan diet in terms of greenhouse gas emissions (e.g., kg CO_2_ equivalent per day or per calorie). Vegan diets had a daily carbon footprint of 1.38 kg CO_2_-eq in a Polish study [97] and 2.6 versus 5.3 kg CO_2_-eq/day in an Icelandic study [98], compared with higher values for omnivorous diets. A US study found that the vegan diet generated 0.69 kg CO_2_ equivalent per 1000 kcal, which was lower (*p* < 0.05) than the pescatarian (1.66), omnivore (2.23), paleo (2.62), or keto (2.91) diets [69].

Regarding O_2_, a recent study [99] compared the cardiovascular fitness of nine habitual vegan with sixteen habitual omnivorous young, healthy men by assessing the relative and absolute VO_2_max on a cycle ergometer. The data indicated no difference between groups for both relative and absolute VO_2_max.

Blancquaert and colleagues [100] assigned 40 healthy female omnivores to either an omnivorous group (*n* = 10), a vegetarian group that was supplemented with creatine and-alanine (*n* = 15), or a vegetarian group that received a placebo (*n* = 15) over a period of six months. At baseline, 3 months, and 6 months, the subjects performed an incremental cycling test to assess VO_2_max. VO_2_max did not differ between groups at baseline, nor did it change during the 6-month intervention period.

Hietavala and co-authors conducted a cross-over design study with nine healthy recreationally active men [101]. Subjects were assigned to both the low-protein vegetarian and the omnivorous diet for four days each. After the low-protein vegetarian diet, VO_2_ was significantly higher at 40% (*p* = 0.035), 60% (*p* < 0.001), and 80% (*p* < 0.001) of VO_2_max compared to the omnivorous diet.

Another study was carried out on patients with type 2 diabetes [102]. In this study, 37 participants were assigned to a hypocaloric (500 kcal) vegetarian or hypocaloric omnivorous diet group. Both groups performed aerobic exercises three times a week for 12 weeks. The results revealed an increase in VO_2_max by 12% (*p* < 0.001) in the vegetarian diet group but no significant changes in the omnivorous diet group.

#### 3.2.5. Intermittent Fasting

Intermittent fasting is not about what you eat, but when you eat it, such as 16 h of fasting followed by 8 h of eating [103]. The diet focuses on timing rather than food choices. By restricting calories and allowing the body to switch between burning glucose and fat for energy, the diet promotes metabolic flexibility. This increases insulin sensitivity and reduces blood sugar spikes. During fasting periods, the body can increase autophagy (cellular repair) [104] and improve mitochondrial efficiency [105]. The diet can be combined with other diets (such as the Mediterranean or ketogenic diets) for additional metabolic benefits.

In a study on rats subjected to intermittent fasting (24 h fasting periods for 1, 7, or 15 days), regional brain glutamate levels were measured. Glutamate levels were found to be significantly reduced in the midbrain, thalamus/hypothalamus, and hippocampus after one day of dieting but returned to the baseline or fluctuated with prolonged fasting [106]. These results cannot be extrapolated to systemic levels due to compartmentalized metabolism (e.g., splanchnic sequestration of dietary glutamate) [107].

An untargeted metabolomic analysis of human blood during 58 h of fasting identified 44 metabolites that increased significantly, including TCA cycle-related metabolites [108]. An approximately two-fold increase in 2-oxoglutarate was observed.

Despite the lack of data on pyruvate during intermittent fasting, there are results from fasting. Pyruvate shows a consistent decrease during water-only fasting (with *p* < 0.01 on days 3 and 5 of fasting) [109], and a significant decrease in pyruvate is observed after Ramadan fasting (*p* < 0.019) [110]. 

Direct measurements of ATP, NADH, and NADP in humans are lacking in the available literature. The hypothesis paper suggests that intermittent fasting may increase NAD levels by activating AMPK, increasing NAMPT expression, or improving the NAD/NADH ratio [111]. However, these are mechanistic speculations not supported by direct experimental measurements. A study on mice subjected to a 24 h fasting showed an increase in liver NAD levels and NAMPT activity, but this was a single prolonged fasting, not intermittent fasting [112].

Other hypothesis papers suggest that intermittent fasting may influence NADPH levels through ketosis-induced citrate export. Fasting increases mitochondrial export of citrate, which is formed from ketone bodies and is metabolized in the cytoplasm by isocitrate dehydrogenase 1 (IDH1) to form NADPH [113]. Studies in aged mice have not found significant changes in the NADPH in the liver cytoplasm with dietary restriction [114].

For CO_2_ and O_2_, there are only indirect measurements obtained during fasting. Numerous studies have measured the respiratory exchange ratio (RER) during fasting, which reflects the ratio of CO_2_ produced to O_2_. A 60 h fasting study found lower RER (closer to 0.7) during fasting [115]. A 21-day fasting study reported that the RER tended toward fat metabolism (decreasing to ~0.7), indicating reduced CO_2_ production relative to O_2_ consumption [116]. An 8-week time-restricted feeding (TRF) study noted a significant decrease in respiratory ratio (equivalent to RER) in the TRF group, suggesting enhanced fat oxidation [117]. A large study using the Lumen device (a breath analyzer) measured the percentage of CO_2_ in an exhaled breath after fasting and found that longer fasting was associated with lower levels of % CO_2_ in an exhaled breath [118].

Based on available data (Table 3), only the Mediterranean diet offers the most direct human evidence for stable glutamic acid levels. It is also associated with a decrease in pyruvate, which is linked to its documented health benefits. A similar effect can be expected from the keto diet and fasting, but these remain largely hypothetical due to a lack of direct human data. For other BMS—including oxoglutarate, NADH, NADP, and ATP—direct measurements in humans are still lacking. However, the Mediterranean diet, with its balanced nutrient profile and proven health benefits, suggests that it supports BMS levels through overall metabolic balance.

Regarding CO_2_, all diets affect its excretion from the body, but differently. A US study found that the vegan diet had the lowest CO_2_ equivalent per calorie (0.69 kg/1000 kcal), significantly lower than the pescatarian (1.66), omnivore (2.23), paleo (2.62), or keto (2.91) diets [69]. While this study did not include the Mediterranean diet, separate data indicates it is also a lower-impact option. Adherence to the Mediterranean diet was associated with lower odds of high dietary CO_2_ emissions, with a dose-response relationship showing progressively lower odds for higher emission quartiles [67]. The moderate metabolic activity associated with this diet is consistent with data showing a corresponding level of oxygen consumption.

Based on the available evidence, the Mediterranean diet emerges as the most justified choice for the baseline diet. This conclusion rests on three key points: First, it is the only diet reviewed with direct human evidence showing stable glutamate levels and a beneficial reduction in pyruvate. Second, its environmental impact is moderate, associated with lower CO_2_ emissions than several other diets, which aligns with physiological data on efficient metabolic activity (reflected in O_2_ consumption). Finally, while direct human data for other BMS are lacking, the diet’s proven health benefits and balanced nutrient composition provide a strong indirect argument for its ability to support overall metabolic equilibrium, in contrast to other, more extreme diets.

### 3.3. Foods Selected to Increase the Level of BMS

To modify the baseline diet for elevating BMS levels in the organism, one can select foods enriched with BMS or their metabolic precursors. This is consistent with the core tenet of nutritional science, which holds that food composition is essential for diet development and assessment [51]. However, given the specific nature of this work, this principle was extended by explicitly incorporating metabolic knowledge. We propose a framework where understanding metabolic connections—such as precursor relationships, enzymatic cofactors, and regulatory effects—informs the selection of foods to translate metabolomic insights into dietary instructions.

#### 3.3.1. Glutamic Acid

Glutamic acid is a non-essential amino acid found in high-protein foods. Animal proteins and fermented foods are particularly rich sources of this amino acid. The source of such animal proteins can be eggs, chicken, beef, and fish (for example, salmon and tuna). Plant proteins as a source of glutamine are found in soybeans, lentils, chickpeas, and spirulina. Among fermented foods, miso, tempeh, and soy sauce can be distinguished [119,120].

#### 3.3.2. Oxoglutaric Acid

Oxoglutaric acid is a key metabolite of the Krebs cycle (Figure 3). Its level depends on the availability of precursors and cofactors involved in metabolism. Glutamate is the main source of nitrogen for the synthesis of oxoglutaric acid [121], and glutamate is deaminated with the formation of this acid. Products rich in glutamine and glutamate [122], such as meat (beef, chicken), fish (salmon, tuna), eggs, dairy products (cottage cheese, cheese), spinach, parsley, tofu, and cabbage, can increase the level of oxoglutaric acid. Also, the level of oxoglutaric acid is affected by foods such as whole grains, nuts (almonds, walnuts), seeds (sunflower, chia), legumes, liver, and eggs, which are rich in B vitamins that act as cofactors for the enzymes of the Krebs cycle.

Products rich in magnesium, such as spinach, cashews, almonds, bananas, and dark chocolate, accelerate the conversion of oxoglutaric acid into the following metabolites, as magnesium activates α-ketoglutarate dehydrogenase [123]. Citrus fruits (oranges, lemons), kiwi, bell peppers, broccoli, and avocado contain antioxidants (vitamin C and glutathione), which protect mitochondria from oxidative stress, maintaining the efficiency of the Krebs cycle and the production of oxoglutaric acid. Products with citric acid (lemons, limes, oranges, and grapefruits) support the overall speed of the cycle since they contain citric acid—the starting substrate of the Krebs cycle.

#### 3.3.3. Pyruvic Acid

Since pyruvate is a product of glycolysis, foods rich in complex carbohydrates will provide the glucose needed for its production. Whole grains best suited for this are: oats, barley, quinoa (provide glucose for glycolysis), legumes (lentils, chickpeas, black beans provide complex carbohydrates and protein), fruits (apples, berries, bananas (natural sugars for glycolysis)), and dairy products (yogurt, kefir).

#### 3.3.4. ATP

ATP levels depend on its production and are determined by the efficiency of glycolysis, the Krebs cycle, and oxidative phosphorylation. Foods rich in magnesium, B vitamins, and healthy fats optimize these processes. The best foods for this are fatty fish, such as salmon, mackerel, and sardines (rich in omega-3 for mitochondrial health); whole grains: quinoa, brown rice, and oats (provide complex carbohydrates for glycolysis); nuts and seeds: almonds, walnuts, and chia seeds (rich in magnesium, a cofactor for ATP synthesis [124]); and leafy greens, such as spinach and kale (rich in magnesium and iron).

#### 3.3.5. NADH

While a high-fat/sugar diet causes energy overload, culminating in reduced NAD/NADH ratio [125] and decreased NAD levels [126,127], the level of NAD can be increased by products rich in B vitamins [128] and complex carbohydrates, which will enhance its production in glycolysis and the Krebs cycle. Suitable for this are fatty fish, which support the function of mitochondria (salmon, mackerel, sardines), meat by-products rich in B vitamins (liver, kidneys), whole grains (quinoa, oats, barley; provide glucose for glycolysis), and legumes (lentils, chickpeas; complex carbohydrates and B vitamins).

#### 3.3.6. NADP

NADP is critical for anabolic reactions and antioxidant protection [129]. Foods rich in niacin (vitamin B3) and folate support its synthesis. Such foods include leafy greens (spinach, kale, and arugula, which are rich in folate and antioxidants), nuts and seeds (almonds, sunflower seeds) rich in niacin, and whole grains rich in B vitamins (brown rice and whole wheat). Among animal products, niacin-rich proteins from chicken, turkey, and fish are suitable.

#### 3.3.7. CO_2_

CO_2_ is produced as a result of cellular respiration. Foods that fuel glycolysis and the Krebs cycle increase CO_2_ production as part of energy metabolism [130]. As previously stated, carbohydrate-rich foods provide fuel for glycolysis and the Krebs cycle (whole grains, fruits, vegetables), and protein-rich foods (eggs, fish, legumes) support amino acid metabolism.

#### 3.3.8. O_2_

Efficient oxygen utilization depends on hemoglobin (Ferrum) levels and, conversely, the severity of oxidative stress. Ferrum-rich foods (spinach, red meat, lentils, tofu) support hemoglobin production [131]. In addition, nitrate-rich foods (beets, arugula, celery) improve blood flow and oxygen delivery.

Table 4 summarizes top foods that have been selected for inclusion in the baseline diet for elevating BMS in the body.

### 3.4. Food Supplements Selected to Increase the Level of BMS

To increase the levels of BMS in the body, specific food supplements can be used. These supplements provide BMS, their precursors, cofactors, or substrates for related biochemical reactions. Table 5 contains a list of supplements and their mechanisms of action.

### 3.5. Scientific Evidence Supporting the Direction and Safety of BMS Level Changes

To confirm the proposed modulation of BMS levels in the direction of increase and to assess the associated risk, an analysis of relevant scientific data was conducted.

A 2024 double-blind, placebo-controlled randomized clinical trial investigated the tolerability of glutamine supplementation in older adults (mean age 77 years) [138]. Participants received a daily dose of 12.4 g of oral glutamine for 60 days. The study concluded that this dosage was well tolerated and safe, with no adverse effects reported, supporting its potential as a viable intervention for maintaining health in aging individuals.

A state-of-the-art review summarizes the biological effects of oxoglutaric acid from a healthy aging perspective [139]. The review highlights that blood concentrations of oxoglutaric acid can decrease as much as 10-fold with age (from 40 to 80 years). Supplementing with oxoglutaric acid has been shown in various in vivo and in vitro studies to positively influence protein synthesis and absorption, bone structure and strength, age-related muscle loss and weakness, and cholesterol regulation.

Decreased ATP levels are a key marker of aging, leading to impaired cell regeneration and the development of age-related diseases. Two recent human studies provide evidence for ATP’s role in healthy aging. A 10-month randomized study on middle-aged individuals (45–72 years) showed that intervention with a functional food significantly increased red blood cell ATP levels [140]. This was associated with a counteraction of age-related endothelial dysfunction, redox dysregulation, and bioenergetic decline. Two human clinical trials presented in 2025 demonstrated that supplementing with a patented form of ATP (PEAK ATP^®^) significantly enhanced amino acid absorption from dietary protein [141]. This improved protein bioavailability is crucial for maintaining muscle mass and supporting metabolic health in older adults. Additionally, oral ATP administration has been shown to prevent exercise-induced declines in ATP and its metabolites, while enhancing peak power and muscular excitability [142].

With age, the level of pyruvic acid in the blood and tissues tends to decrease, while lactate content increases, indicating metabolic shifts and a possible decline in the efficiency of tissue respiration [143]. Chronic pyruvate supplementation increases exploratory activity and brain energy reserves in young and middle-aged mice [144]. Today, pyruvate is being considered as an alternative to popular anti-aging drugs (NAD precursors and senolytics), as it may act as a substitute for them. Scientists suggest that pyruvate-enriched fluids (e.g., oral rehydration salt solutions) could become a novel intervention for age-related diseases. However, direct evidence of pyruvate’s benefits for healthy aging is currently limited, and further intensive research is needed [145]. A 2024 review emphasizes that the long-term effects of pyruvate supplementation in healthy individuals (e.g., on physical performance) have not been confirmed in well-controlled studies [146].

A decrease in NAD levels is also a key marker of aging, and increasing its levels is currently one of the key anti-aging strategies [147,148]. NADP(H) levels also decline with age due to a decrease in the overall activity of NAD(P)-dependent enzymes and a decline in energy metabolism, which is accompanied by a reduced ability of cells to resist oxidative stress and impaired tissue repair. Currently, directly increasing NADP levels is not used as an anti-aging treatment. However, this is not a limiting factor for our metabolomics-guided nutrition, as it does not directly supplement with this substance (Table 3).

With age, oxygen consumption and carbon dioxide production decrease due to reduced muscle mass, slower metabolism, and decreased elasticity of lung tissue [149]. Increased oxygen consumption (VO_2_) and carbon dioxide production (VCO_2_) are physiologically beneficial for the body (excluding pathologically related hypercapnia). This is observed during beneficial aerobic exercise and the EPOC (excess post-exercise oxygen consumption) state, which restores energy reserves, enhances protein synthesis, and utilizes metabolic byproducts (e.g., lactate) [150]. Increased oxygen consumption activates cognitive activity, as the brain is the primary consumer of oxygen. An increase in carbon dioxide levels (within reasonable limits) indicates an increase in “waste release,” enhances oxygen release by hemoglobin into tissues (the Bohr effect), and acts as a natural vasodilator, improving blood flow.

Thus, the strategy of the proposed nutrition aimed at increasing BMS levels corresponds to the modern scientific concepts and can be implemented safely for humans.

### 3.6. Evaluation of Nutrition Effeciency

#### 3.6.1. Age-Related Metabolomic Curve

**Figure 5:**
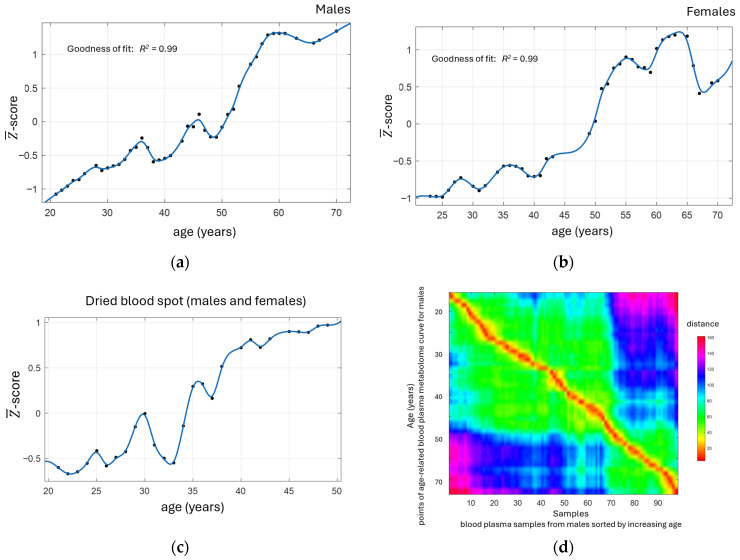
Age-related dynamics of blood plasma metabolome demonstrated by metabolomic fingerprinting ($\bar{Z}$-score curves). (**a**) For male blood plasma samples; (**b**) for female blood plasma samples; (**c**) for male and female dried blood spot (DBS) samples; (**d**) matrix of distances between metabolic fingerprints of men’s blood plasma fingerprints and age-related metabolic curve points. $\bar{Z}$-score is the mean of the intensities of age-correlated mass spectrometric peaks, represented as Z-score. *R*^2^—coefficient of determination for curve fitting.

Direct mass spectrometry was selected as the most straightforward method for obtaining blood metabolome fingerprints, which were processed to obtain the age-related metabolomic curves ($\bar{Z}$-score versus age) for both genders (Figure 5a–c).

The figure visualizes the nonlinear, age-related dynamics of the metabolome. Changes occur in distinct phases or “waves”. After 50 years in men and 47 years in women, more intense near-linear changes lead to fluctuations in the metabolome at a new level. Notably, the nonlinear dynamics of the metabolome with age and the timing of the waves are consistent with recently published data [151,152]. Missing values in the mass spectrometry data and moderate variations in the curve construction parameters did not affect its shape (Figures S1 and S2).

The superposition of the individual’s metabolome fingerprints on all points of the age-related metabolomic curve (Figure 5d) shows that, despite the presence of a wave in the $\bar{Z}$-score curve, the metabolome fingerprints have age specificity, which allows them to be used for biological age determination across the entire age range.

Figure 5c shows that the age-related metabolomic curve for the DBS samples differs slightly from that obtained with plasma, but the overall waveform is preserved.

#### 3.6.2. Biological Age Change Determination

**Figure 6:**
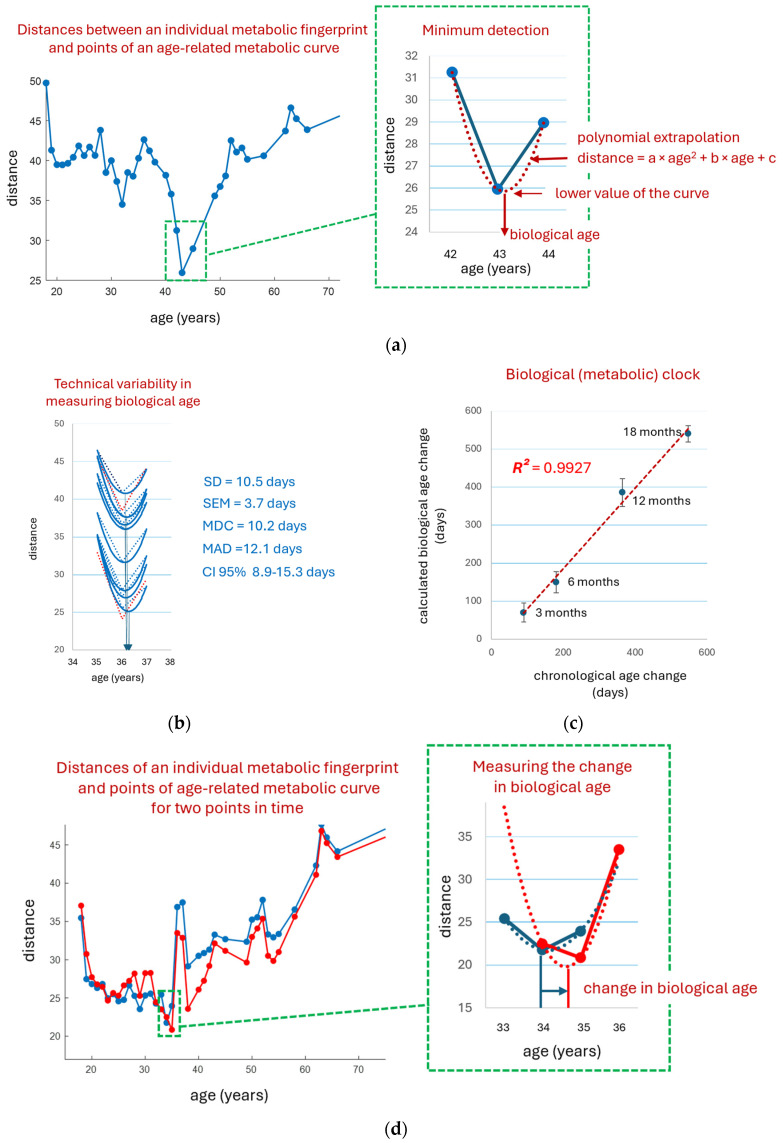
Biological age change determination using individual metabolome fingerprint and age-related metabolomic curve. (**a**) The distances (Euclidean distance) from an individual’s metabolic fingerprint to all points on the age-related curve. Where the distance is minimal, the distance curve indicates the biological age of the individual. The right panel shows extrapolation of three points forming the minimum of the distance curve for a more accurate determination of the biological age of an individual. (**b**) Result of biological age determination for technical replicates of mass spectrometric measurements. Red dashes indicate outliers not included in the calculation of technical variability parameters. (**c**) Comparison of measurements of change in biological age with change in chronological age (“metabolic clock”). *R*^2^—coefficient of determination for line extrapolation. (**d**) An example of biological age change calculation as the difference in its values between two time points (red and blue colors).

Measuring the change in an individual’s biological age using an age-related metabolomic curve is proposed in the most simplified way. The distance (Euclidean distance) from an individual’s metabolic fingerprint to all points on the curve is measured. Where the similarity is greatest, the distance is minimal, indicating the biological age of the individual. An example of such a distance measurement is shown in Figure 6a. Given that the age-related curve is constructed with a resolution of 1 year, extrapolation from three points forming the minimum was used to accurately determine biological age (Figure 6b). The technical variability of this approach is quite low, and the MAD is only 12 days (CI 95% 8.9–15.3) (Figure 6b). Figure 6c confirms the accuracy of biological age measurements, as they are linearly related to changes in chronological age (the *R*^2^ coefficient of determination for linear extrapolation was 0.99). Figure 6d shows an example of measuring biological age change as the difference in its values between two time points.

**Figure 7:**
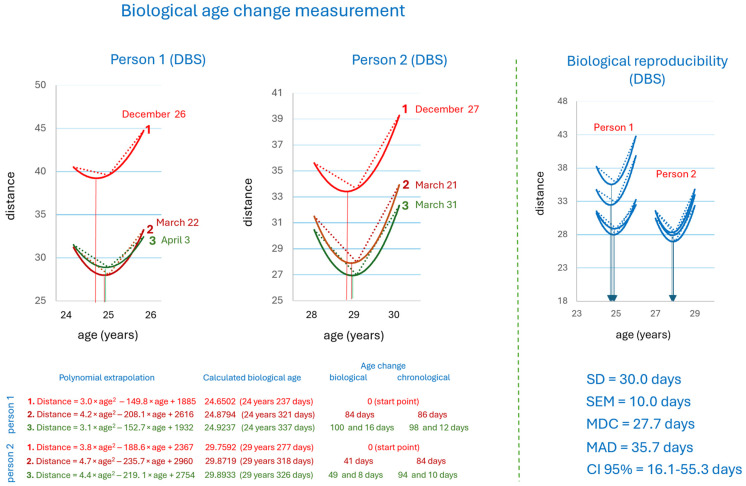
An example of measuring biological age change using DBS samples. The dotted line corresponds to the minimum distance of the individual’s metabolomic fingerprint to the age-related metabolomic curve. The solid line represents the extrapolation of this minimum over three points, the lower value of which indicates biological age. The first time point is marked in red, the following ones in brown and green. The right panel shows the measurement of biological age to determine biological reproducibility using DBS samples.

Figure 7 shows an example of implementing this approach for DBS of two individuals, each with a long (3-month) and two following short-term sample collections. The measurements were characterized by different precision, indicating interindividual variability, likely related to individual metabolome properties, since the measurements were performed identically.

### 3.7. Food Intake Influence on the Biological Age Measurement

**Figure 8:**
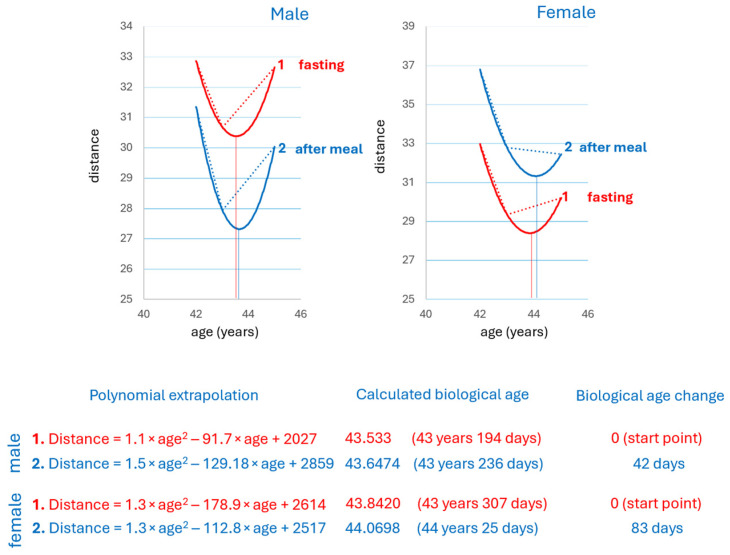
The influence of food intake on biological age determination. Red color indicates fasting measurements, blue indicates one hour after eating. The dotted line shows the minimum of the distance curve between the individual metabolome fingerprint and the age-related metabolomic curve. The solid line is a three-point extrapolation of the minimum, the lower value of which indicates biological age.

Given the profound influence of food on the blood metabolome, the extent of food’s influence on biological age determinations was assessed. Figure 8 shows that food intake has a moderate effect, close to biological variability. This feature is characteristic of the measurement method, which confirms the need to use fasting blood samples.

## 4. Discussion

### 4.1. Scientific Evidence Linking BMS to Health and Longevity

The summary of the involvement of BMS in metabolic processes and their relation to disease (Figure 3) shows that the main processes involving BMS occur in mitochondria [153]. Oxoglutaric acid (α-ketoglutarate) and pyruvic acid (pyruvate) occur there as key intermediates in the Krebs cycle (citric acid cycle). Glutamic acid, a precursor of oxoglutaric acid, links amino acid metabolism to the Krebs cycle. ATP is the end product of oxidative phosphorylation that occurs in mitochondria. ATP is the main energy currency of the cell, and its decline is associated with aging [154]. NAD is a key electron carrier in the electron transport chain that helps generate ATP. Decreased NAD levels and associated mitochondrial dysfunction are also associated with aging-related diseases [155]. NADP and its reduced form NADPH are critical for combating oxidative stress [156]. NADPH participates in the regeneration of antioxidants, such as glutathione, which protect cells from damage. Oxygen is needed to produce ATP and is a source of reactive oxygen species (ROS), which contribute to aging and aging-related diseases [157]. CO_2_ is a byproduct of the Krebs cycle that is eliminated from the body. Dysregulation of CO_2_ levels can affect pH [158] and cell function [159], contributing to aging.

Scientific evidence for the involvement of BMS in aging-related processes (Figure 4) confirms that metabolomics-derived BMS are integrated the in main aging-related events in the organism. Thus, the BMS-centric design of healthy and anti-aging nutrition is not only metabolomically sound but also justified in terms of accumulated today scientific evidence.

### 4.2. Selection of a Baseline Diet for Precision Nutrition

To implement precision nutrition, a baseline diet must be selected for subsequent adjustment based on metabolic response. For this purpose, we focused on widely recognized diets, as their extensive literature allows for a systematic assessment of their effects on BMS. Based on the available evidence, the Mediterranean diet emerges as the most justified choice for the baseline diet (Table 3). It is important to note that the availability of scientific data on it and the information gaps regarding other, less popular diets influence the choice. This implies that as additional information becomes available, the baseline diet will be modified. Nevertheless, for now, its selection as the baseline diet can be considered well founded.

### 4.3. Foods Selected to Increase the Level of BMS

Data on the specific changes in BMS concentrations following food consumption are scarce. This gap exists because such precise measurements—for example, tracking blood level changes after ingestion—are typically applied to pure, well-characterized substances like pharmaceuticals to determine pharmacokinetic parameters. In contrast, foods are complex, unstandardized mixtures of numerous compounds that interact with one another. Furthermore, their composition is highly variable, and they are consumed as part of a mixed meal, leading to significant variations in the bioaccessibility and bioavailability of their constituents [160]. The only possible option in this case is to simply use information on the composition of foods and the known scheme for their use. Following this principle, foods enriched with BMS or their metabolic precursors were suggested to modify the baseline diet for elevating BMS levels in the organism (Table 4). The list of these foods can be supplemented with foods with known composition, for example, from FoodData Central (https://fdc.nal.usda.gov, accessed on 22 March 2026 ) [119] or the Food Database (FooDB; https://foodb.ca) [120].

For baseline dietary change, the overarching scheme, represented by the Nutrition Care Process (NCP), which is a standardized model used by registered dietitians, is proposed. Medical history, current health status, anthropometrics (height, weight, BMI), dietary intake history, food preferences, cultural practices, socioeconomic status, and psychological factors are considered. It should also be noted that the uncontrolled use of food supplements leads to their excess in the body, which may cause side effects and dangerous interactions with medications, as well as toxicity, a significant healthcare issue [161,162,163,164].

### 4.4. Implementation of Metabolomic-Guided Precision Nutrition

**Figure 9:**
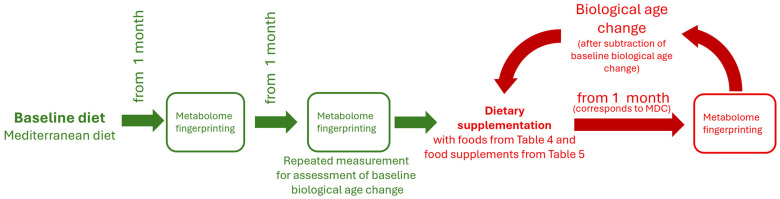
Concept of metabolomics-guided healthy and anti-aging precision nutrition. The dietary protocol begins with a baseline (Mediterranean) diet, which has minimal impact on BMS levels. This baseline diet is then dynamically adjusted in response to changes in the individual’s metabolic age, which is precisely measured using metabolome fingerprinting. Based on this assessment, the diet is progressively tailored by incorporating specific foods and supplements designed to modulate BMS levels. The periods of measurement of changes in biological age are determined by the minimum detectable change (MDC) in biological age, which was calculated from experimental data.

Precision nutrition involves providing dynamic dietary recommendations tailored to individual metabolic characteristics. In this study, recommendations are based on metabolic age (biological age estimated from metabolic data) and, more specifically, its change in response to dietary intervention. To implement this precision nutrition framework, the baseline diet (Mediterranean diet) is proposed to be dynamically adjusted according to individual change in the metabolic age (Figure 9). Depending on the individual’s response, the diet is step-by-step personalized by incorporating foods relevant to BMS (from Table 4) and specific food supplements (from Table 5).

### 4.5. Evaluation of Nutritional Efficiency

**Figure 10:**
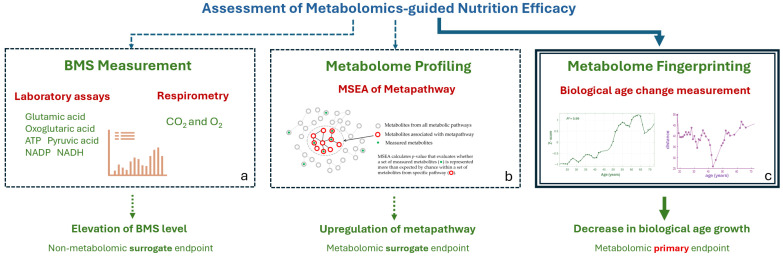
The ways to evaluate efficacy of metabolomics-guided nutrition. The proposed concept of anti-aging precision nutrition includes three options for assessing individual efficacy. Assessing the metapathway state through either: (**a**) measuring biomarkers of metapathway state (BMS) levels or (**b**) metabolite set enrichment analysis (MSEA) based on metabolome profiling. Both are related to surrogate endpoints and are often impractical for routine use due to their indirect relationship with age and, in the case of MSEA, high analytical complexity. (**c**) Metabolome fingerprinting—a direct and comprehensive method that measures the primary outcome of interest: a change in metabolic age. This is the most consistent and promising approach for directly evaluating the slowing of aging.

Since the proposed nutrition is aimed at improving health and slowing down aging by influencing the metapathway, the metapathway state can provide surrogate endpoints of such nutrition. To assess the metapathway state, two approaches can be distinguished. The first is to measure BMS, as they are representative of the metapathway (Figure 10a). Non-volatile BMS can be measured by laboratory assays. Oxygen and CO_2_ can be assessed indirectly by respirometry. Since the measurement of individual substances for evaluating the diet does not involve metabolomics, can be achieved through laboratory assays, and concerns surrogate endpoints, this approach was not explored in this study.

In general, BMS deviations from normal levels are not expected. If such deviations are observed, they may be associated with pathology or other factors, which would likely require consultation with a physician to correct their levels rather than the use of antiaging nutrition. Otherwise, the use of surrogate endpoints appears appropriate for: (i) confirming the mechanism of the diet’s antiaging effect (metabolomic fingerprinting data are confirmed by changes in BMS levels); (ii) identifying the cause of the diet’s ineffectiveness (a negative metabolomic fingerprinting result is combined with no change in BMS levels); and (iii) monitoring BMS levels to prevent them from exceeding the normal range in response to excessive antiaging nutrition, which could have negative health consequences.

To assess the state of the entire metapathway (normal state, upregulated, downregulated), it is possible to use metabolite set enrichment analysis (MSEA) (Figure 10b). In metabolomics, enrichment analysis is extremely common. However, the implementation of the MSEA for a large metabolic pathway, such as the metapathway, is problematic. MSEA requires the identification and accurate quantification of many metabolites by metabolome profiling of blood plasma. Such an analysis is carried out by a group of scientists specializing in metabolomics and, in terms of time and cost, is related to a full-fledged scientific study, which seems unjustified for a routine assessment of nutrition. Therefore, this approach is mentioned here as an impractical but well-described available option.

The last option, metabolomic fingerprinting, represents the most promising approach for assessing the nutritional outcome (Figure 10c). Since the metapathway was originally identified through multiple untargeted (panoramic) metabolomic analyses, employing a fingerprint as a comprehensive metabolomic metric is a consistent choice. Furthermore, within the framework of anti-aging precision nutrition, metabolomic fingerprinting directly evaluates the anti-aging effect by measuring the primary endpoint—change in metabolic age—unlike the surrogate endpoints discussed above.

The fingerprinting approach was also selected for its practical advantages. A fingerprint is defined as multivariate characteristics derived from the set of mass spectrometric peak intensities. This makes fingerprinting a form of metabolome mining that does not require metabolite identification. By forgoing metabolite identification, the method is radically simplified. Furthermore, utilizing the combined signal from many metabolites allows to buffer fluctuations in any single metabolite; this averaging process generates a stable and robust composite signal.

Traditionally, biological age measurements are validated by chronological age. In our case, we used the change in chronological age, as it is closer to assessing biological age in response to dietary changes. Figure 6c validates biological age measurements by metabolomic fingerprinting, as they are linearly related to changes in chronological age (*R*^2^ = 0.99).

The translational nature of the study makes it appropriate to evaluate DBS samples for biological age measurements. DBS is more complementary to dietary adjustments, as blood samples are collected at home, stored, and transported to the laboratory in a dried form at room temperature. An assessment of biological variability of biological age determination by metabolic fingerprinting yielded an MDC of 27.7 days for using DBS (Figure 7), which corresponds to the precision of the measurement. Therefore, the anti-aging effect of the diet can be recorded if it consists of a change in biological age of one month or more. In such a situation, it is advisable to monitor biological age change using DBS no more than once a month. It is noteworthy that the obtained MDC value does not contradict the larger MAD value obtained for biological reproducibility. The MDC is the smallest change in a measurable value that reliably exceeds random measurement error or system “noise”. MAD is a measure of the dispersion of data around a center (usually the mean) and characterizes the variability of the data itself.

It should be noted that the obtained precision for biological age measurement cannot be considered fully representative, as it depends not only on the properties of the blood metabolome but also on mass spectrometry (sensitivity of the used mass spectrometer, the number of detected mass peaks, the quality of mass spectra processing, and feature extraction options). In the case of routine use of metabolome analysis, the degree of influence of biological confounders (see Section 4.6) and how they were mitigated will also affect the reproducibility of the data. The reproducibility parameters measured in this study should be considered as an achievable reference, which may vary for the worse in further human tests, or, perhaps, for the better with targeted efforts. 

### 4.6. Biological Confounders in Metabolomics-Guided Nutrition

The proposed metabolomics-guided nutrition must account for biological factors that influence outcomes, especially those that affect blood metabolome. Previous research has shown that genetics, gut microbiome, clinical parameters, nutrition, lifestyle, and anthropometric measurements collectively explain more than 76% of the variance in blood metabolites [165]. Among these, nutrition and microbiome exert the strongest influence, accounting for over 50% of the observed metabolite variation. In the context of an intervention for an individual, the influence of genetics can be considered constant and ignored. Similarly, lifestyle and anthropometric factors are expected to remain stable during short-term dietary modifications. 

The pronounced influence of food on the blood metabolome prompted us to evaluate the impact of food on biological age determination. It was found that food intake has a moderate effect, close to biological variability (Figure 8). This may be due to the multiparametric nature of the metabolome fingerprint, which smooths out fluctuations in individual metabolites. To avoid such influence, it seems sufficient to take fasting blood samples for measurements.

Since nutrition and microbiome are primary determinants of blood metabolome, the biological age response must be carefully considered whenever dietary adjustments are made. It is advisable to monitor biological age dynamics following dietary adjustments until a new steady-state value is reached. The true effect of nutritional intervention on biological age should then be assessed from this stabilized point. Notably, because foods are complex mixtures with varying nutrient bioavailability, and due to individual differences in intestinal absorption, metabolism, and initial microbiome composition, the lag time between dietary change and metabolome stabilization is expected to vary across individuals. Generalizable patterns regarding the biological age change and the time required to reach a new steady state can be established through further human tests of the proposed nutritional framework.

### 4.7. Limitations

In this work, the metabolomic datasets on human aging and disease were translated by focusing on a limited set of central metabolites within the metapathway. These metabolites, i.e., BMS, were selected based on their high centrality rank. However, the optimal number of metabolites for metabolomics-guided nutrition remains an open question; a smaller set might simplify dietary guidance without losing efficacy, while a larger one could improve it. Our final selection was guided by the availability of scientific data on their role in biochemical processes and aging, the existence of human measurement techniques, and information on their presence in foods and supplements.

Regarding the baseline diet and recommendations, no restrictions are foreseen other than the well-known ones, such as the presence of diseases requiring special nutrition, elderly people, and the data presented being applied to adults. In addition, the usefulness of the Mediterranean diet for healthy longevity is well known and can be recommended to a wide range of people. This work further supports its usefulness based on metabolomic data.

Among the limitations of the presented nutrition is its reliance on supplementation with foods and food supplements to personalize the baseline diet for an anti-aging effect. However, it is possible that, based on human testing, an anti-aging effect will be observed with a reduction in BMS levels. In this case, it is recommended to reduce the consumption of the foods listed in Section 3 and Table 4 if they have been included in the baseline diet.

The influence of gut microbiota on the precision of biological age measurement remains an important and unaccounted factor. A previous study using an identical protocol for blood metabolomic fingerprinting by DIMS demonstrated the significant impact of changes in even individual groups of gut microbiome microorganisms on the results [166]. Therefore, a separate study linking changes in the gut microbiome to the precision of biological age measurement using blood metabolomic fingerprinting appears highly relevant in the near future.

The conceptual nature of this study dictated limited testing of metabolomic fingerprinting as a means of monitoring dietary effectiveness. The tests confirmed its efficacy, but with a limited number of samples. Specifically, the DBS sample set did not include elderly individuals, and biological reproducibility was studied using samples only from middle-aged individuals. Although the test results support the concept of metabolomics-based nutrition, questions remain about its effectiveness in older adults, since the impact of confounders in older individuals may differ significantly. For example, the gut microbiota, which influences the blood metabolome, exhibits significant differences in older individuals and is characterized by a decrease in beneficial microorganisms and an increase in opportunistic microorganisms (so-called “age-related dysbiosis”). Therefore, the effectiveness of metabolomic fingerprinting in older adults as a means of monitoring the proposed nutrition requires further research.

The key limitation concerns metabolomic control and subsequent adjustment of the proposed diet. Although the accuracy of the biological age determination from the metabolome is measured in years [167], it can precisely estimate the *change* in biological age within an individual. This approach is most precise, since it excludes the influence of interindividual biological variability.

### 4.8. Final Notes

A distinctive feature of the proposed nutrition is its use of accumulated scientific metabolomic data on diseases and aging, which has not previously been observed in diets or other metabolomics-based nutritional approaches. This is the main achievement of this study and makes the proposed precise nutrition new. Furthermore, the precise assessment of biological age change for assessing the effectiveness of nutrition also has not previously been used.

The long-term significance of this study is largely determined by the ability to objectively measure the health benefits of diet, individual foods, and food supplements. This may ultimately lead to a renewed understanding of their healing properties and the development of new, more effective diets.

The next phase of research is a T2-stage translational study (translation to patients; Figure 1). This phase aims to evaluate the efficacy and effectiveness of the proposed nutritional intervention in the target population and to inform evidence-based guidelines. Specifically, this stage will:

- Confirm the translational potential of the underlying scientific basis;
- Clinically validate proposed nutrition (assess its strength, reproducibility, and ability to account for biological confounders);
- Test, refine, or adjust the accuracy of measuring changes in biological age using metabolome fingerprints in response to dietary modifications;
- Identify practical limitations;
- Evaluate the overall feasibility of implementing the proposed nutrition, considering its labor intensity and the cost of implementation.

## 5. Conclusions

The role of nutrition in health, healthy aging, and longevity is undeniable. However, both dietary practices and scientific understanding of them continually evolve. Consequently, a pressing challenge is the translation of the latest scientific discoveries into actionable nutritional strategies for health improvement. The recent identification of a unified metapathway underlying aging and disease provided a translational foundation for this modernization. The existence of a limited number of representative BMS for the overall metapathway enabled the design of BMS-centric dietary instructions, forming the basis for metabolomics-guided precision nutrition. The proposed nutrition is now ready for the next phases of translation, which include developing evidence-based guidelines and establishing its effectiveness in humans.

## Figures and Tables

**Table 1:** Metabolites with the highest centrality (central nodes) in the metabolite–metabolite interaction network constructed for the metapathway. Adapted from [40].

Metabolite Name	Degree (Connections of Node)
L-Glutamic acid	57
Oxoglutaric acid	53
Adenosine triphosphate (ATP)	51
Pyruvic acid	50
NADP	46
CO_2_	46
NADH	44
O_2_	40
On average for all metabolites in the network	
	24

**Table 2:** Characteristics of the sample sets used in this study.

Set of Samples	Age (Years)	Subjects (Samples)	Gender (Male/Female)	Sample Type	Purpose of Set of Samples
Set 1	18–81	190 (190)	96/94	Blood plasma	To build and characterize age-related metabolic curve.
Set 1.1	36	1 (10)	1/0	Blood plasma	For technical variability calculation.
Set 2	18–66	124 (124)	51/72	Blood plasma	To build age-related metabolic curve for the “metabolic clock” test.
Set 2.1	18–54	15 (30)	6/9	Blood plasma	For testing biological age calculation by comparing with chronological age (“metabolic clock” test).
Set 3	18–81	100 (100)	60/40	DBS	To build age-related metabolic curve for DBS samples.
Set 4	25, 29	2 (6)	0/2	DBS	To demonstrate the measurement of biological age change at different time intervals.
Set 5	25, 29	2 (7)	0/2	DBS	To calculate the biological reproducibility of biological age change measurement.
Set 6	43, 43	2 (4)	1/1	DBS	To demonstrate the influence of food intake on the measurement of biological age.

**Table 3:** Summary of available data on the effects of diets on biomarkers of metapathway state (BMS).

BMS	Diet				
	Mediterranean	Ketogenic	Paleolithic	Vegan	Intermittent Fasting
Glutamic Acid	No change (direct) [59]	No change (direct, in CSF of epilepsy patients) [71]; increase (indirect, theoretical) [72]	No data; expected stable (indirect) [87]	Increase (direct) [110]	Decrease (indirect, in specific brain regions in rats) [106]; fluctuates (indirect) [106]
Oxoglutaric Acid	No data	Fluctuates (indirect, theoretical) [76]	No data; expected stable (indirect)	Expected stable (indirect)	Increase (direct) [108]
ATP	Increase (indirect, theoretical) [61,63]	Increase (indirect, in rodent brain) [78]; drops then increases (indirect, theoretical) [77]	Increase (indirect, theoretical) [88]	Increase (indirect, theoretical) [96]	No data
Pyruvic Acid	Decrease (direct) [60]	Decrease (indirect, theoretical)	Expected stable (indirect)	Expected stable (indirect)	Decrease (direct) [109,110]
NADH	Increase (indirect, theoretical) [77,78]	NAD/NADH ratio increase (direct, in human brain) [80]	No data; expected stable (indirect)	No data	Increase (indirect, theoretical) [111]
NADP	No data; indirect support of function (indirect) [65]	NADP/NADPH ratio decrease (indirect, theoretical) [81,82,83]	No data; indirect influence (indirect) [89,90]	Expected high (indirect) [89,90]	No change (indirect, in aged mice) [114]; increase (indirect, theoretical) [113]
CO_2_ emission	Lower dietary emissions (direct) [66,67]	Decrease (direct) [84,85]	No data	No data on physiological levels; lower dietary emissions (direct) [69,97,98]	Decrease (indirect, via RER) [115,116,118]
O_2_ (VO_2_max)	Increase (direct) [68,69]	No data	No data	No change (direct) [99,100]; increase (direct, at submaximal levels) [101]; increase (direct, in diabetic patients) [102]	No data; fat oxidation increases (indirect, via RER) [115,116,117]

BMS: biomarkers of metapathway state. Direct: data obtained from human studies. Indirect: data from animal studies, theoretical assumptions, or inferred from related metabolic effects. CSF: cerebrospinal fluid. RER: respiratory exchange ratio (CO_2_ produced/O_2_ consumed). A lower RER indicates higher fat oxidation.

**Table 4:** Foods selected for elevating biomarkers of metapathway state (BMS) levels.

BMS	Foods
Glutamic Acid	Eggs, chicken, soybeans.
Oxoglutaric Acid	Citrus fruits, spinach, almonds, liver.
ATP	Salmon, quinoa, almonds, spinach.
Pyruvic Acid	Oats, lentils, apples, yogurt.
NADH	Salmon, liver, quinoa, lentils.
NADP	Spinach, almonds, brown rice, chicken.
CO_2_	Whole grains, fruits, eggs, legumes.
O_2_	Spinach, red meat, blueberries, beets.

BMS: biomarkers of metapathway state.

**Table 5:** Food supplements selected for elevating biomarker of metapathway state (BMS) levels.

BMS	Food Supplements	Mechanisms of Action
Glutamic acid	L-glutamine	A precursor to glutamic acid, supporting amino acid metabolism and neurotransmitter synthesis.
	Whey protein	Rich in glutamine and glutamic acid [132].
	Spirulina	A plant-based source of glutamic acid and other amino acids [133].
Oxoglutaric acid	Oxoglutaric acid	Directly supplements oxoglutaric acid.
	B-vitamin complex	Enhance the oxoglutaric acid metabolism by supporting the Krebs cycle by providing cofactors (e.g., B1, B2, B3, B5).
	Magnesium	Enhance the oxoglutaric acid metabolism providing a cofactor (magnesium) for enzymes in the Krebs cycle.
ATP	CoQ10	Supports mitochondrial ATP production [134].
	D-ribose	A sugar that serves as a backbone for ATP synthesis [135].
	Creatine monohydrate	Enhances ATP regeneration, especially in muscle cells [136].
	Magnesium	A cofactor for ATP synthesis and utilization.
Pyruvic acid	Calcium pyruvate	Directly supplements pyruvate, supporting glycolysis and energy production.
	B-vitamin complex	Supports glycolysis and the conversion of pyruvate to acetyl-CoA.
	Magnesium	A cofactor for enzymes in glycolysis.
NADH	Nicotinamide riboside	Increases NAD levels, which are converted to NADH in energy metabolism.
	Nicotinamide mononucleotide	A precursor to NAD, supporting NADH production.
	B-vitamin complex	Provides cofactors for NADH production in glycolysis and the Krebs cycle.
NADP	Niacin (vitamin B3)	A precursor for NADP synthesis.
	Nicotinamide riboside	Elevate NADP levels by increasing NAD availability.
	Folate (vitamin B9)	Supports NADP-dependent reactions in anabolic pathways.
CO_2_	Bicarbonate supplements	Support CO_2_ buffering and transport in the blood.
	Citrate	Supports the Krebs cycle and CO_2_ production.
	B-vitamin complex	Enhances metabolic pathways that produce CO_2_.
O_2_	Ferrum	Supports hemoglobin production, improving oxygen transport.
	CoQ10	Enhances mitochondrial function and oxygen utilization.
	Beetroot powder	Rich in nitrates, which improve blood flow and oxygen delivery [137].
	Antioxidants (vitamin C and E)	Reduce oxidative stress, improving oxygen efficiency.

BMS: biomarkers of metapathway state.

## Data Availability

The data presented in the study are openly available in FigShare at https://doi.org/10.6084/m9.figshare.29591708.

## Supplementary Materials

The following supporting information can be downloaded at: https://www.mdpi.com/article/10.3390/metabo16040241/s1, Figure S1: Age-related dynamics of the blood plasma metabolome represented by $\bar{Z}$-score curves constructed under different feature extraction conditions. Figure S2: The percentage of mass spectrometry peaks with non-zero intensity values used to construct the $\bar{Z}$-score curve of age-related metabolome dynamics. Table S1: Characteristics of blood sample donors (Set 1). Table S2: Characteristics of blood sample donors (Set 3). Table S3: Characteristics of blood sample donors (Set 2). Table S4: Characteristics of blood sample donors (Set 2.1).

## Abbreviations

The following abbreviations are used in this manuscript:

MSEA	Metabolite set enrichment analysis
BMS	Biomarker(s) of metapathway state
ATP	Adenosine triphosphate
NAD(P)	Nicotinamide adenine dinucleotide (phosphate)
MAD	Mean absolute difference
MDC	Minimum detectable change
CI 95%	95% confidence interval

## Appendix A

### Appendix A.1. Participation of Biomarkers of Metapathway State (BMS) in Various Aging-Related Processes

#### Appendix A.1.1. L-Glutamic Acid

1. Antioxidant Support

- Glutamic acid is a precursor to glutathione, one of the body’s most important antioxidants. Glutathione neutralizes reactive oxygen species (ROS) and reduces oxidative stress, a primary driver of cellular aging and the pathogenesis of age-related diseases [168].
- By supporting glutathione synthesis, glutamic acid indirectly helps protect cellular components (including DNA, proteins, and lipids) from oxidative stress throughout life [168].

2. Detoxification

- Glutamic acid is involved in the synthesis of glutathione, which plays a key role in detoxifying harmful substances, including heavy metals and environmental toxins. Efficient detoxification helps maintain cellular health and reduces the risk of age-related damage [168].

3. Energy Metabolism

- Glutamic acid is a key anaplerotic agent, meaning it can be converted into α-ketoglutarate to replenish the Krebs cycle. This is vital for sustaining energy production, particularly in tissues with high metabolic rates. A decline in mitochondrial function is a hallmark of aging, and supporting metabolic flux through the Krebs cycle is therefore critical [169].
- By contributing to the production of ATP, the primary energy currency of cells, glutamic acid supports fundamental cellular processes, helping to maintain vitality and reduce fatigue associated with aging [169].

4. Neurotransmitter Function

- Glutamic acid is the predominant excitatory neurotransmitter in the human central nervous system (CNS). It is essential for synaptic plasticity, which underpins learning and memory—cognitive functions that often decline with age [169,170].
- Disturbances in the glutamate–glutamine cycle, often secondary to other age-related conditions like hepatic impairment, can compromise memory function and contribute to encephalopathies [170].
- Glutamic acid serves as the precursor for the synthesis of gamma-aminobutyric acid (GABA), the main inhibitory neurotransmitter. The proper balance between excitatory (glutamate) and inhibitory (GABA) neurotransmission is critical for preventing neuronal hyperexcitability and excitotoxicity, which are implicated in age-related neurodegenerative diseases [169].

5. Protein Synthesis and Cellular Repair

- As an amino acid, glutamic acid is a building block for proteins, which are essential for cellular repair and regeneration. Adequate protein synthesis is necessary for maintaining tissue integrity and function, especially as the body ages [169].

6. Immune System Support

- Immune cells, such as lymphocytes and macrophages, rely heavily on glutamine (synthesized from glutamate) as a metabolic fuel, particularly during activation. A robust immune system, capable of responding effectively to pathogens and malignancies, is a key feature of healthy aging [171].
- Glutamine availability influences the proliferation of immune cells and the production of antibodies. During catabolic states common in severe illness or advanced age, glutamine can become conditionally essential for optimal immune function [171].

7. Ammonia Detoxification

- The brain is highly susceptible to ammonia toxicity. Glutamic acid, through the enzyme glutamine synthetase, plays a non-negotiable role in detoxifying ammonia in the brain by converting it into glutamine. This process is vital for preventing neurotoxicity [170,172].
- Impaired liver function, which becomes more common with age, can lead to hyperammonemia. The glutamine synthetase reaction, dependent on glutamate, is a critical defense mechanism against ammonia-induced neurological damage, helping to maintain cognitive health [170,172].

Key findings from human research:

Ref. [168]: Supplementation with precursor amino acids (cysteine, glycine, glutamate) enhances tissue glutathione synthesis more effectively than direct glutathione supplementation in humans.

Refs. [169,170]: Glutamate is the primary excitatory neurotransmitter in the human CNS, and disturbances in its homeostasis are directly linked to cognitive deficits in conditions like hepatic encephalopathy.

Ref. [171]: In critical illness (a model of accelerated aging), glutamine is routinely supplied in clinical nutrition protocols to support metabolic and immune function. In HIV+ patients with depleted levels, glutamine supplementation (20 g/day) helped normalize plasma glutathione levels, highlighting its conditional essentiality in immunocompromised states.

Ref. [172]: Hepatic glutamine synthetase is responsible for a significant portion (approx. 35%) of the body’s ammonia detoxification capacity in humans, underscoring its systemic importance.

#### Appendix A.1.2. Oxoglutaric Acid (α-ketoglutarate, α-KG)

1. Energy Production

- As a critical component of the Krebs cycle, α-KG is essential for ATP production, the primary energy currency of cells. Efficient energy metabolism is crucial for maintaining cellular function and preventing age-related decline [173,174].
- By supporting mitochondrial function, α-KG helps ensure that cells have the energy needed for repair and maintenance. Mitochondrial dysfunction is a hallmark of aging, and the activity of the α-ketoglutarate dehydrogenase complex (KGDHC), which utilizes α-KG, is severely reduced in age-related neurodegenerative diseases like Alzheimer’s [175].

2. Antioxidant Defense

- α-KG is involved in the synthesis of glutathione, a major antioxidant that protects cells from oxidative stress. Oxidative stress is a key factor in aging and age-related diseases [176].
- By promoting glutathione production, α-KG helps neutralize reactive oxygen species (ROS) and reduce cellular damage. Research in human erythrocytes (red blood cells) has demonstrated that α-KG can serve as a superior glutamate source for the synthesis of glutathione compared to glutamine, especially when provided at certain concentrations [176]. A study on long-livers (aged 90–102) found their erythrocytes were characterized by elevated levels of glutathione and related amino acids, contributing to a superior anti-oxidative stress capacity [177].

3. Amino Acid Metabolism

- α-KG serves as a precursor for the synthesis of several amino acids, including glutamate and glutamine. These amino acids are important for protein synthesis, cellular repair, and overall metabolic health [173,174].
- Proper amino acid metabolism is essential for maintaining tissue integrity and function as we age. The glutamine-α-KG metabolism is pivotal in nitrogen and ammonia balance, which is crucial for metabolic health [176].

4. Collagen Synthesis

- α-KG is a cofactor for prolyl hydroxylase, an enzyme necessary for the synthesis of collagen. Collagen is a structural protein that maintains skin elasticity, joint health, and overall tissue strength [173].
- Supporting collagen production can help reduce wrinkles, improve skin texture, and maintain joint flexibility, contributing to a more youthful appearance. Clinical applications note its role in promoting wound healing [178].

5. Epigenetic Regulation

- α-KG is a cofactor for enzymes involved in epigenetic regulation, such as the TET (ten-eleven translocation) family of enzymes and histone demethylases. These enzymes play a role in DNA demethylation and chromatin remodeling, which are important for gene expression and cellular differentiation [173,179].
- Proper epigenetic regulation is crucial for maintaining cellular identity and function, and its dysregulation is associated with aging and age-related diseases [180]. This mechanistic link is the basis for ongoing human clinical trial testing if α-KG supplementation can reduce DNA methylation age, a biomarker of biological aging [180].

6. Detoxification

- α-KG is involved in the urea cycle, which helps detoxify ammonia by converting it into urea for excretion. Reducing ammonia levels is important for preventing cellular damage and maintaining overall health [174].
- Efficient detoxification processes are essential for reducing the burden of toxins that can accelerate aging. This role is well documented in clinical settings, where AKG is used to control uremia in hemodialysis patients [178].

7. Immune System Support

- α-KG plays a role in immune cell function and regulation. It can influence the differentiation and activity of immune cells, helping to maintain a balanced and effective immune response [173,174].
- A robust immune system is important for protecting against infections and diseases that become more prevalent with age. As a precursor to glutamine, α-KG supports immune system function and protein metabolism [173].

8. Longevity Pathways

- α-KG has been shown to influence longevity pathways, such as the mTOR (mechanistic target of rapamycin) and AMPK (AMP-activated protein kinase) pathways. These pathways regulate cell growth, metabolism, and survival [179].
- Modulation of these pathways can promote cellular health and extend lifespan. Preclinical models show AKG inhibits mTOR and activates AMPK [179]. A direct link to human longevity was found in a 2024 study, which discovered that long-livers (age 90+) exhibit a unique “youthful” metabolic reprogramming in their erythrocytes, enabling better oxygen release and antioxidative capabilities, which are critical for combating age-related decline [177]. Furthermore, a 2023 review highlighted AKG as a potent regulator of both healthspan and lifespan [179].

Key findings from human research:

Ref. [176]: Glutamine and α-ketoglutarate as glutamate sources for glutathione synthesis (in vitro study on human erythrocytes).

Ref. [177]: Long-livers have youthful erythrocyte function (human study of centenarians’ erythrocyte metabolism).

Ref. [179]: Alpha-ketoglutarate as a potent regulator for lifespan and healthspan (comprehensive review of evidence and mechanisms).

Ref. [180]: Alpha-ketoglutarate supplementation and biological age (ABLE) trial protocol (human RCT protocol measuring DNA methylation age).

#### Appendix A.1.3. Adenosine Triphosphate

1. Energy Supply for Cellular Functions

- ATP provides the energy required for essential cellular processes, including DNA repair, protein synthesis, and cell division. Efficient energy supply is crucial for maintaining cellular function and preventing age-related decline.
- By ensuring that cells have sufficient energy, ATP supports the repair and maintenance of tissues and organs, which is vital for longevity. A core theory in geroscience posits that maintaining or increasing energy metabolism and ATP levels is essential for promoting the survival of older animals, as it fuels the energy-intensive repair and homeostatic mechanisms that prevent cellular aging [181].

2. Mitochondrial Function

- ATP is produced in mitochondria through oxidative phosphorylation. Healthy mitochondrial function is essential for sustained energy production and for reducing the accumulation of cellular damage. The heart, for instance, requires a continuous and rapidly adjustable supply of ATP to meet energetic demands, and an age-related decrease in maximal myocardial oxygen consumption and cardiac efficiency is observed, suggesting a deterioration in ATP supply-to-demand matching [182].
- Mitochondrial dysfunction is a hallmark of aging. In skeletal muscle, aging is associated with a lower oxidative capacity, but intriguingly, older muscle adapts by utilizing oxidative ATP production at a greater percentage of its capacity rather than increasing non-oxidative pathways, indicating a form of bioenergetic rigidity [183].

3. DNA Repair

- ATP is required for the activity of enzymes involved in DNA repair, such as DNA ligases and polymerases. Efficient DNA repair mechanisms are crucial for maintaining genomic stability and preventing mutations that can lead to cancer and other age-related diseases. DNA damage is a major internal factor leading to genomic instability and is a key driver of the aging process [5].
- By supporting DNA repair, ATP helps protect cells from the cumulative damage that contributes to aging. The continuous accumulation of DNA-damaged cells triggers cell death and senescence, ultimately leading to chronic inflammation and loss of function [5].

4. Protein Homeostasis

- ATP is necessary for the proper folding and degradation of proteins through processes like chaperone-mediated folding and proteasome activity. Maintaining protein homeostasis (proteostasis) is important for preventing the accumulation of misfolded or damaged proteins, which is associated with aging and neurodegenerative diseases [5,184].
- By supporting proteostasis, ATP helps maintain cellular function and reduce the risk of age-related protein aggregation diseases. The loss of protein balance is recognized as a key characteristic of aging [5].

5. Cellular Detoxification

- ATP is required for the function of ATP-binding cassette (ABC) transporters and other detoxification mechanisms that remove harmful substances from cells [185]. Efficient detoxification helps protect cells from damage and supports overall health.
- Reducing the burden of toxins can slow the aging process. Notably, aging significantly reduces the protein expression of key ABC transporters like P-glycoprotein (P-gp) and Breast Cancer Resistance Protein (BCRP) in the BBB of mice, even though their mRNA levels remain unchanged. This age-related decline in detoxification capacity could elevate the risk of neurotoxicity and central nervous system adverse drug reactions in the elderly [185].

6. Cell Signaling and Communication

- ATP is involved in cell signaling pathways, including those mediated by kinases and G-protein-coupled receptors. Proper cell signaling is essential for coordinating cellular responses to stress, nutrients, and growth factors [186,187].
- Effective communication is crucial for homeostasis. Extracellular ATP is a potent signaling molecule that modulates immune and inflammatory responses. Its breakdown product, adenosine, primarily exerts anti-inflammatory effects, creating a delicate balance in immune regulation. Dysregulation of this balance is implicated in age-related chronic inflammation [188].

7. Muscle Function and Mobility

- ATP is essential for muscle contraction and relaxation. Maintaining adequate ATP levels supports muscle function, strength, and mobility, which are important for quality of life as we age [187].
- By supporting muscle health, ATP helps prevent sarcopenia (age-related muscle loss) and maintains physical independence. Research in C. elegans has shown that compounds like febuxostat (FBX), which can increase ATP levels, help protect mitochondria and prevent age-related muscle deterioration, suggesting a direct link between cellular energy availability and muscle integrity during aging [189].

8. Neuroprotection

- Neurons require a significant amount of ATP to maintain their function, including synaptic transmission and ion gradient maintenance. The brain is the highest consumer of ATP in the body, consuming approximately twenty-five percent of the total energy available. A large amount of this energy is spent on maintaining ion concentrations for proper neuronal signaling and synaptic transmission [187].
- Adequate ATP levels are crucial for cognitive health. By supporting neuronal energy demands, ATP helps protect against age-related cognitive decline. The age-related reduction in ABC transporter function within the BBB could also lead to increased accumulation of neurotoxic compounds, further highlighting the need for robust ATP-dependent detoxification in the aging brain [185].

9. Anti-Inflammatory Effects

- ATP can modulate immune responses and reduce inflammation when present in appropriate amounts or contexts. Chronic inflammation (inflammaging) is a key driver of aging [5,184].
- By supporting anti-inflammatory processes, ATP contributes to health. The conversion of pro-inflammatory extracellular ATP to anti-inflammatory adenosine by ectonucleotidases (CD39/CD73) is a critical immunoregulatory mechanism. This pathway helps resolve inflammation and maintain immune balance, and its dysregulation with age can contribute to a persistent pro-inflammatory state [188].

Primary human-based and clinically relevant studies:

Ref. [5]: Comprehensive review of molecular mechanisms of aging. The review shows that DNA damage is a major driver of aging.

Ref. [183]: Bioenergetic rigidity in older skeletal muscle (human study comparing ATP production pathways in young and older adults).

Ref. [185]: Aging reduces ABC transporter protein expression in the blood–brain barrier (study on brain microvessels from young (12-week) and aged (85-week) mice).

Ref. [188]: Extracellular ATP and adenosine balance immune responses (review article discussing the role of purinergic signaling in inflammation).

#### Appendix A.1.4. Pyruvic Acid

1. Energy Production

- Pyruvic acid is a crucial link between glycolysis and the Krebs cycle. It is converted into acetyl-CoA, which enters the Krebs cycle to produce ATP, the primary energy currency of cells [190,191].
- Efficient energy production is essential for maintaining cellular function and preventing age-related decline. A key study using hyperpolarized ^13^C-MRI in healthy human adults (*n* = 35, ages 21–77) demonstrated a significant age-associated decline in the conversion of pyruvate to acetyl-CoA (measured as ^13^C-bicarbonate production) in the brain, at a rate of approximately −9% ± 4% per decade. This provides direct in vivo evidence in humans of reduced mitochondrial pyruvate metabolism with aging [192].

2. Antioxidant Properties

- Pyruvic acid has been shown to have antioxidant properties, helping to neutralize reactive oxygen species (ROS) like hydrogen peroxide (H_2_O_2_) through a non-enzymatic decarboxylation reaction, producing water, carbon dioxide, and acetate [193].
- By reducing oxidative damage to cellular components like DNA, proteins, and lipids, pyruvic acid helps protect cells from aging-related damage. This direct scavenging activity has been demonstrated in in vitro models using human cells [193]. Furthermore, its role in supporting NADPH production via the pentose phosphate pathway indirectly bolsters cellular antioxidant defense systems [194].

3. Cellular Repair and Regeneration

- Pyruvic acid is a precursor for alanine and other amino acids, which are blocks for proteins. Adequate protein synthesis is necessary for cellular repair and regeneration [195].
- By supporting protein synthesis, pyruvic acid helps maintain tissue integrity and function, which is crucial for slowing aging. Dysregulation of this anaplerotic replenishment of biosynthetic precursors is a feature of age-related metabolic decline.

4. Detoxification

- Pyruvic acid is involved in the metabolism of lactate, helping to convert lactate back into pyruvate, which can then be used for energy production. This process helps reduce lactic acid buildup, which can cause cellular damage and fatigue [191,195].
- Efficient lactate metabolism supports cellular health and reduces the risk of age-related metabolic dysfunction. The heart, for example, can utilize lactate as a fuel source, converting it back to pyruvate [196]. Age-related shifts in this balance can contribute to metabolic inflexibility.

5. Neuroprotection

- Pyruvic acid can cross the blood–brain barrier and be used as an energy source for neurons. Adequate energy supply is crucial for maintaining cognitive function and preventing neurodegenerative diseases [197].
- By supporting neuronal energy demands, pyruvic acid helps protect against age-related cognitive decline. Computational models of the aging brain indicate that impaired metabolic support, including perturbations in pyruvate-derived substrates, disrupts neuronal ATP production and electrical activity, which are hallmarks of brain aging [197]. The aforementioned ^13^C-MRI study also found a significant age-related decrease in the conversion of pyruvate to lactate (anaerobic glycolysis) in most brain regions, further highlighting a broad decline in cerebral pyruvate metabolism with age [192].

6. Anti-Inflammatory Effects

- Pyruvic acid, particularly in the form of ethyl pyruvate, has been shown to have anti-inflammatory properties in vivo and in vitro, helping to reduce chronic inflammation (inflammaging) by inhibiting key pro-inflammatory pathways like NF-κB and HMGB1 release [193].
- By modulating inflammatory responses, pyruvic acid helps protect tissues and organs from damage and supports overall health. These effects have been observed in models of lung disease and exposure to toxicants, suggesting a broad anti-inflammatory potential relevant to aging. [193,198].

Primary human-based or clinically relevant studies cited:

Ref. [192]: Human study using hyperpolarized ^13^C-MRI in 35 adults showing decreased brain pyruvate metabolism with age. Study shows age-associated change in pyruvate metabolism.

Ref. [193]: Review detailing the direct ROS scavenging mechanism of pyruvate, supported by in vitro human cell studies.

Ref. [197]: Computational model integrating human data, showing the critical role of pyruvate-derived energy for neuronal function in aging.

#### Appendix A.1.5. NADP(H)

1. Antioxidant Defense

- NADP is essential for maintaining the reduced form of glutathione (GSH), a key antioxidant in cells. The enzyme glutathione reductase (GR) uses NADPH to convert oxidized glutathione (GSSG) back to GSH in a critical redox cycle [199]. This reaction is fundamental for sustaining the cell’s reducing environment, as the ratio of GSSG/GSH is a key indicator of cellular oxidative balance [200].
- GSH helps neutralize reactive oxygen species (ROS), which are byproducts of metabolism that can damage cellular components like DNA, proteins, and lipids. By reducing oxidative stress, NADP helps protect cells from aging-related damage. The importance of this system is highlighted in conditions like cystic fibrosis, where impaired GSH secretion leads to increased oxidative damage and inflammation in the lungs [199,200].

2. Cellular Repair

- NADPH supports the synthesis of nucleotides, which are necessary for DNA replication and repair. This occurs primarily through the pentose phosphate pathway (PPP), which generates both NADPH and ribose-5-phosphate, a pentose sugar essential for nucleotide biosynthesis.

3. Energy Metabolism and Mitochondrial Function

- NADP is involved in the pentose phosphate pathway (PPP), which generates NADPH and ribose-5-phosphate. NADPH is crucial for reductive biosynthesis and protecting cells from oxidative damage.
- Healthy mitochondrial function is essential for energy production and reducing age-related decline. NADPH helps maintain mitochondrial integrity by mitigating oxidative stress within the organelle. The mitochondrial NADP pool is distinct and essential for sustaining the activity of mitochondrial antioxidant systems, including glutathione and thioredoxin pathways. A decline in the ability to generate mitochondrial NADPH is linked to increased oxidative damage and dysfunction in aging [65].

4. Sirtuin Activation

- NADP is indirectly linked to sirtuins, a family of proteins involved in regulating cellular health and longevity. Sirtuins require NAD^+^ (a related molecule) for their activity, and the balance between NAD^+^ and NADPH is critical for cellular homeostasis [147,201].
- Sirtuins play a role in DNA repair, inflammation reduction, and metabolic regulation, all of which are important for slowing aging. The age-related decline in cellular NAD^+^ levels directly impairs sirtuin activity, contributing to metabolic syndrome, mitochondrial dysfunction, and accelerated aging. Strategies to boost NAD^+^ levels have been shown to improve mitochondrial function and healthspan in both model organisms and humans, demonstrating the profound interconnection between NAD(H) and NADP(H) pools in aging biology [147,201].

5. Redox Balance

- The NADP/NADPH couple helps maintain the cellular redox balance, which is crucial for preventing oxidative damage and ensuring the proper functioning of metabolic pathways. Disruption of redox balance is a hallmark of aging [65].
- Importantly, an excess of reducing equivalents (NADPH and GSH) can lead to a state of reductive stress, which is as detrimental as oxidative stress. Reductive stress can disrupt metabolic signaling, promote pathological hypertrophy, and contribute to protein aggregation diseases by inhibiting necessary oxidative folding steps. This highlights the critical need for a precise redox balance, rather than a simple surplus of antioxidants, for healthy aging [65].

6. Anti-Inflammatory Effects

- NADPH is involved in the production of nitric oxide (NO) by nitric oxide synthase (NOS), which has anti-inflammatory and vasoprotective effects. All three NOS isoforms (NOSI, NOSII, NOSIII) require NADPH as a cofactor to synthesize NO from L-arginine [202,203].
- Chronic inflammation (inflammaging) is a key driver of aging. NO helps regulate immune responses and maintain vascular health. However, it is crucial to note that in a state of oxidative stress, NOS can become “uncoupled,” leading to the production of superoxide instead of NO, which paradoxically exacerbates inflammation and oxidative damage [203]. This uncoupling is a significant feature of endothelial dysfunction in aging humans. Furthermore, clinical studies provide evidence that insufficient NO production is associated with all major cardiovascular risk factors and has profound predictive value for the progression of cardiovascular and Alzheimer’s disease [203].

Primary human-based or clinically relevant studies and reviews cited:

Ref. [199]: Comprehensive review detailing the central role of GSH and its NADPH-dependent regeneration in cellular defense.

Ref. [65]: Review expanding the concept of redox imbalance to include the pathological consequences of reductive stress.

Ref. [201]: Major review discussing the age-related decline in NAD^+^ and its impact on sirtuins and downstream processes like mitochondrial function.

Ref. [203]: Review focusing on the decline in NO production with human aging and its link to age-related diseases.

#### Appendix A.1.6. NADH

1. Energy Production

- NADH is a key player in cellular respiration, particularly in the electron transport chain (ETC) in mitochondria. It donates electrons to Complex I of the ETC, which drives the production of ATP through oxidative phosphorylation [204].
- Efficient ATP production is essential for maintaining cellular function and preventing age-related decline in energy metabolism. A direct correlation between the brain’s NAD^+^/NADH redox ratio and the rate of ATP production has been demonstrated in humans using ^31^P-MRS, indicating that a favorable redox state is crucial for sustaining energy metabolism, especially in the energy-demanding brain [205].

2. Mitochondrial Function

- NADH is critical for the proper functioning of mitochondria. The transfer of electrons from NADH to the ETC is a primary source of the proton gradient necessary for ATP synthesis [204].
- Mitochondrial dysfunction is a hallmark of aging. Importantly, it is not just the loss of ATP production but also the associated reductive stress caused by a low NAD^+^/NADH ratio that contributes to pathology. Genetic tools that increase the NAD^+^/NADH ratio have been shown to ameliorate metabolic and proliferative defects caused by an impaired ETC, underscoring the critical role of redox balance in mitochondrial health [206].

3. Antioxidant Defense

- NADH provides reducing equivalents for biosynthetic pathways and redox defense. However, its reduced counterpart, NADPH, is the primary cofactor for regenerating antioxidants like glutathione. The NAD^+^/NADH and NADP^+^/NADPH systems are distinct but interconnected [207].
- The relationship between NADH and oxidative stress is complex. While NADPH is directly involved in neutralizing ROS, the NAD^+^/NADH ratio influences the activity of sirtuins and other enzymes that regulate the cellular response to oxidative stress. A dysregulated NAD^+^/NADH ratio can thus indirectly contribute to oxidative damage, a key factor in aging [207,208].

4. Sirtuin Activation

- Sirtuins, a family of NAD^+^-dependent deacylases, are crucial regulators of longevity. They require NAD^+^ as a cofactor for their activity, and their function is therefore intrinsically linked to the cellular NAD^+^/NADH ratio [201,209].
- The age-related decline in NAD^+^ levels directly impairs sirtuin activity, contributing to metabolic syndrome, mitochondrial dysfunction, and accelerated aging. Strategies to boost NAD^+^ levels have been shown to improve mitochondrial function and healthspan in model organisms and are being actively investigated in humans [201,207]. It is the availability of NAD^+^, not NADH, that is the limiting factor for sirtuin activation.

5. Cellular Signaling and Communication

- NADH and its precursors are involved in various signaling pathways. For instance, NAD^+^ is a substrate for enzymes like CD38 and PARPs, which generate calcium-mobilizing second messengers and are involved in DNA repair, respectively [209].
- These NAD^+^-consuming signaling pathways are a major source of NAD^+^ depletion during aging. The enzyme CD38, whose expression increases with age, is a significant consumer of NAD^+^ and is considered a primary driver of age-related NAD^+^ decline, thereby disrupting cellular communication and stress responses [201,209].

6. Neuroprotection

- Neurons have a high energy demand, and efficient ATP production is critical for synaptic transmission and ion gradient maintenance. The NAD^+^/NADH redox ratio is a key determinant of mitochondrial energy production in the brain [205].
- By supporting neuronal energy demands, maintaining a healthy a NAD^+^/NADH ratio helps protect against age-related cognitive decline. Human ^31^P-MRS studies have shown that brain NAD levels and the NAD^+^/NADH ratio are positively associated with ATP levels and the rate of energy production, and that these metrics decline with age [205].

7. Anti-Inflammatory Effects

- NAD^+^ metabolism is a key modulator of immune and inflammatory responses. The NAD^+^-consuming enzyme CD38 is highly expressed on immune cells, and its activity influences inflammatory pathways [201,209].
- Chronic inflammation (inflammaging) is a key driver of aging. The age-related increase in CD38 expression not only depletes NAD^+^ pools but also contributes to a pro-inflammatory state. Conversely, boosting NAD^+^ levels can suppress NF-κB signaling and reduce inflammation, highlighting the anti-inflammatory potential of targeting NAD^+^ metabolism [201,207].

Primary human-based or clinically relevant studies cited:

Ref. [205]: Human ^31^P-MRS study in 50 participants linking brain NAD^+^/NADH ratio to ATP production.

Ref. [206]: Study using LbNOX to demonstrate the pathological role of reductive stress (high NADH) in mitochondrial dysfunction.

Ref. [201]: Comprehensive review detailing the age-related decline in NAD^+^ and its impact on sirtuins and downstream processes.

Ref. [209]: Review identifying CD38 as a major NADase and driver of age-related NAD^+^ decline and inflammation.

#### Appendix A.1.7. Carbon Dioxide

1. Bohr Effect

- CO_2_ facilitates the release of oxygen from hemoglobin in the blood to tissues, a phenomenon known as the Bohr effect. This ensures that cells receive adequate oxygen for energy production and metabolic processes. The Bohr effect describes how increased CO_2_ concentrations facilitate oxygen unloading from hemoglobin in tissues, while the Haldane effect describes how oxygenated blood promotes CO_2_ release [210]. Efficient oxygen utilization is essential for maintaining mitochondrial function and reducing oxidative stress, both of which are critical for anti-aging. This mechanism is particularly important in aging individuals who may experience compromised microcirculation and tissue oxygenation.

2. pH Regulation

- CO_2_ plays a key role in maintaining the acid–base balance (pH) in the body. It acts as a buffer, helping to regulate pH levels in the blood and tissues through the bicarbonate buffer system: CO_2_ + H_2_O ⇌ H_2_CO_3_ ⇌ H^+^ + HCO_3_^−^ [210]. Proper pH balance is important for enzymatic activity, cellular function, and overall homeostasis, which can influence aging processes. The respiratory system helps maintain acid–base balance by removing CO_2_, preventing acidosis that can accelerate aging processes. Age-related declines in respiratory function can compromise this regulation, potentially contributing to age-related physiological decline.

3. Cellular Respiration and Energy Production

- CO_2_ is a byproduct of cellular respiration, the process by which cells generate energy (ATP) from glucose through the reaction: C_6_H_12_O_6_ + 6O_2_ → 6CO_2_ + 6H_2_O [210]. Efficient energy production is vital for maintaining cellular health and function. By supporting mitochondrial function and energy metabolism, CO_2_ indirectly helps reduce age-related decline in cellular activity. The continuous production and elimination of CO_2_ reflect metabolic activity, and its dysregulation may signal mitochondrial dysfunction, which is a hallmark of aging.

4. Anti-Inflammatory Effects

- A hallmark of aging is a state of chronic, low-grade inflammation known as “inflammaging” [211]. CO_2_ regulates key cellular processes, particularly those involving mitogen-activated protein kinases (MAPKs). Since MAPKs play a role in inflammation, manipulating CO_2_ levels can therefore influence inflammatory responses [212].

5. Neuroprotection

- CO_2_ can have a calming effect on the nervous system and may help reduce stress and anxiety. Lower stress levels are associated with slower aging and reduced risk of age-related diseases. It may also support brain health by improving blood flow and oxygen delivery to neural tissues. Research has shown that CO_2_ significantly affects neurovascular coupling (the relationship between neural activity and subsequent changes in cerebral blood flow), which is crucial for maintaining cognitive function [213]. The brain’s high metabolic demand for oxygen (~20% of total body oxygen consumption) makes tight regulation of cerebral blood flow by CO_2_ particularly important for preventing age-related cognitive decline [214].

6. Adaptation to Stress

- Mild increases in CO_2_ levels (e.g., through controlled breathing exercises) can stimulate adaptive responses in the body, such as improved respiratory and cardiovascular function. These adaptations can enhance resilience to stress and support overall health, contributing to anti-aging. The body’s chemoreceptor system, which responds to changes in CO_2_ levels, plays a crucial role in these adaptive processes. Central chemoreceptors located near the ventrolateral surfaces of the medulla are particularly responsive to changes in pCO_2_ and pH, triggering compensatory mechanisms that maintain homeostasis [210]. This adaptive capacity may decline with age, making targeted CO_2_ interventions potentially valuable for healthy aging.

Note: Some references are from clinical review articles that summarize evidence from human studies. While not all are primary research articles, they synthesize data obtained from human subjects.

#### Appendix A.1.8. Oxygen

1. Energy Production

- Oxygen is a key component of cellular respiration, particularly in the electron transport chain (ETC) in mitochondria. It acts as the final electron acceptor, enabling the production of ATP through oxidative phosphorylation [204]. The overall reaction culminates in the reduction of oxygen to water, harnessing energy to phosphorylate ADP to ATP.
- Efficient ATP production is essential for maintaining cellular function, repair, and overall vitality, which are crucial for slowing aging. Age-related declines in maximal oxygen consumption (VO_2_max) are closely tied to reduced ATP production and functional capacity [182,215,216].

2. Mitochondrial Function

- Oxygen is necessary for oxidative phosphorylation, the process by which mitochondria generate ATP. Healthy mitochondrial function is vital for sustained energy production and reducing the accumulation of cellular damage [204,217].
- Mitochondrial dysfunction is a hallmark of aging. Hyperbaric oxygen therapy (HBOT) has been shown to reverse aspects of biological aging in humans by lengthening telomeres and reducing senescent cell burden, suggesting that optimized oxygen delivery can preserve mitochondrial integrity and function [216,218].

3. Cellular Repair and Regeneration

- Oxygen supports the synthesis of new cells and tissues by providing the energy needed for cellular repair and regeneration processes. This is particularly important for maintaining the health of rapidly dividing tissues [216].
- Efficient cellular repair mechanisms help reduce the impact of aging. For example, HBOT has been demonstrated to enhance tissue repair and reduce inflammation in conditions like chronic inflammatory response syndrome (CIRS), which shares features with accelerated aging [219].

4. Antioxidant Defense

- While oxygen is essential for life, it can also contribute to the formation of reactive oxygen species (ROS). However, the body’s antioxidant defense systems rely on oxygen to function effectively [217,220].
- Enzymes like superoxide dismutase (SOD) and catalase require oxygen for their activity. Proper oxygen levels help maintain the balance between ROS production and antioxidant defense. Long-lived individuals exhibit enhanced antioxidant capacities, such as elevated glutathione (GSH) production, which is linked to better management of oxidative stress [177].

5. Collagen Synthesis

- Oxygen is required for the hydroxylation of proline and lysine residues in collagen synthesis, a process catalyzed by prolyl hydroxylase and lysyl hydroxylase. Collagen is a structural protein that maintains skin elasticity, joint health, and overall tissue strength. Supporting collagen production can help reduce wrinkles and improve skin texture. HBOT has been shown to improve skin health and reduce aging markers, possibly through enhanced collagen synthesis [216,218].

6. Immune Function

- Oxygen is crucial for the proper functioning of immune cells, such as neutrophils and macrophages, which rely on oxidative bursts to kill pathogens. A robust immune system is important for protecting against infections and diseases that become more prevalent with age [219,221].
- By supporting immune function, oxygen helps maintain overall health. HBOT has been demonstrated to modulate immune responses, reduce chronic inflammation, and improve outcomes in immune-related chronic conditions like Long COVID [219,221].

7. Neuroprotection

- The brain is highly dependent on oxygen for energy production and function. Adequate oxygen supply is crucial for maintaining cognitive health and preventing neurodegenerative diseases [222].
- Oxygen therapy and techniques like HBOT have been explored for their potential to enhance brain function. Studies show that HBOT can improve neurocognitive function, cerebral blood flow, and overall brain health in aging and post-viral conditions. The brain is highly dependent on oxygen for energy production and function. Adequate oxygen supply is crucial for maintaining cognitive health and preventing neurodegenerative diseases [216,218,221].

8. Detoxification

- Oxygen is involved in various detoxification processes, including the oxidation of toxins in the liver by cytochrome P450 enzymes. Efficient detoxification helps protect cells from damage and supports overall health [219].
- Reducing the burden of toxins can slow the aging process. HBOT has been shown to aid in detoxification and reduce toxin-related inflammation in clinical settings [219].

9. Vascular Health

- Oxygen is essential for maintaining vascular health and ensuring proper blood flow to tissues and organs. Adequate oxygenation supports endothelial function and reduces the risk of age-related vascular diseases [177,222].
- By promoting vascular health, oxygen helps maintain tissue perfusion and overall vitality. Long-lived individuals exhibit better erythrocyte function and oxygen release capacity, which supports optimal tissue oxygenation and vascular health [177].

10. Anti-Inflammatory Effects

- Oxygen can modulate inflammatory responses and promote healing. Chronic inflammation is a key driver of aging, and reducing it can help protect tissues and organs from damage [219,221].
- HBOT has demonstrated significant anti-inflammatory effects in human studies, reducing pro-inflammatory cytokines (e.g., IL-1, IL-6, TNF-α) and elevating anti-inflammatory mediators (e.g., IL-10). This modulation is beneficial in age-related chronic conditions [221].

Primary human-based or clinically relevant studies cited:

Ref. [177]: Study of long-livers showing enhanced antioxidant capacity (e.g., glutathione) and reduced oxidative stress. The study shows that long-livers have youthful erythrocyte function and metabolic signatures.

Ref. [221]: Review of HBOT’s anti-inflammatory and immunomodulatory effects in humans.

Ref. [219]: Case study showing HBOT improves immune markers and reduces inflammation.

Ref. [216]: Human clinical trial showing HBOT-induced telomere lengthening and reduced senescent cells. The trial shows that hyperbaric oxygen therapy reverses aging in two key biological clocks.

Ref. [222]: Study on neurovascular coupling and oxygen delivery mechanisms in the brain. Study shows how the brain increases blood flow on demand.
