# Supplementary material for: Towards Metabolomics-Guided Healthy and Anti-Aging Nutrition
Source: Metabolites. 2026 Apr 1;16(4):241. doi: 10.3390/metabo16040241 (PMC13118237; doi:10.3390/metabo16040241)
Supplement: Supplementary file 1 [file metabolites-16-00241-s001.zip › Figure S1 and Figure S2.pdf]

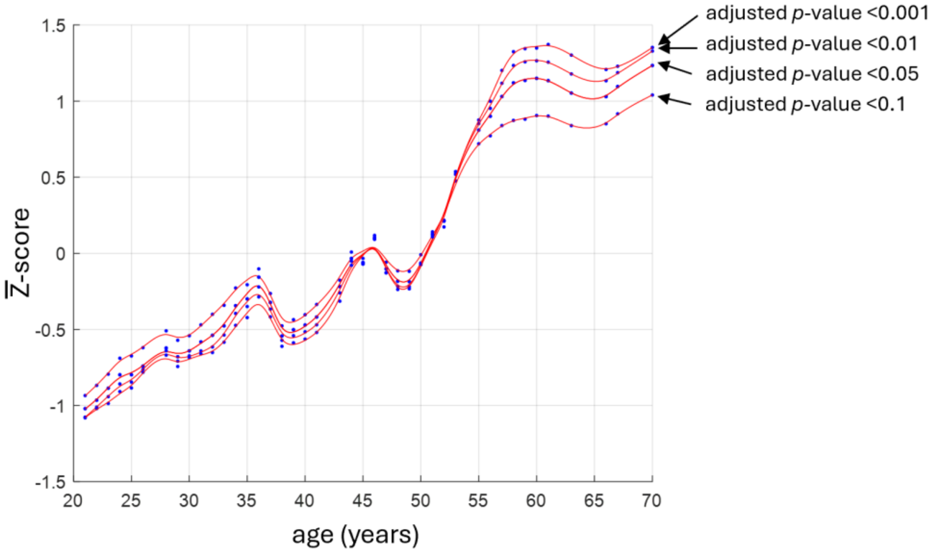

Figure S1. Age-related dynamics of the blood plasma metabolome, represented by $\overline{Z}$-score curves constructed under different feature extraction conditions.

The $\overline{Z}$-score is the mean of the intensities of age-correlated mass spectrometric peaks, expressed as a Z-score. To construct these curves, age-related mass peaks were identified (feature extraction). This was done by calculating probability of a Spearman correlation between peak intensity and age (*p*-values for testing the hypothesis of no correlation against the alternative hypothesis of a nonzero correlation; see Methods section for details). Peaks with significant positive and negative correlations (using different adjusted p-value thresholds) were selected. Their Z-scored intensities were then averaged to generate each $\overline{Z}$-score curve. The figure shows the resulting curves for males from sample Set 1.

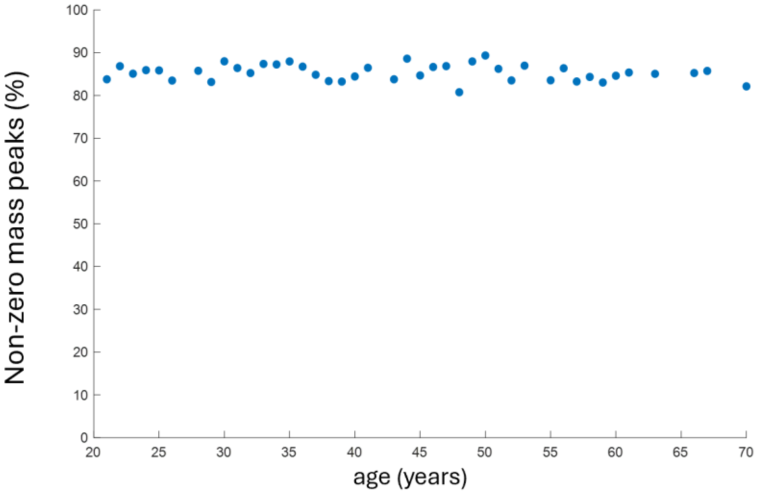

Figure S2. Percentage of mass spectrometry peaks with non-zero intensity values used to construct the $\overline{Z}$-score curve of age-related metabolome dynamics. The figure shows the results for males from sample Set 1.
